# Determining Olefinic and Phenolic Fractions in Dissolved Organic Matter by Ozonation with Stable Oxygen Isotope Analysis of H_2_O_2_

**DOI:** 10.1021/acs.est.6c00499

**Published:** 2026-04-21

**Authors:** Seok Kim, Maria Lia Halder, Thomas B. Hofstetter, Urs von Gunten

**Affiliations:** † 28499 Swiss Federal Institute of Aquatic Science and Technology Eawag, Dübendorf 8600, Switzerland; ‡ Institute of Biogeochemistry and Pollutant Dynamics (IBP), ETH Zürich, Zürich 8092, Switzerland; § School of Architecture, Civil and Environmental Engineering (ENAC), Ecole Polytechnique Fédérale Lausanne (EPFL), Lausanne 1015, Switzerland

**Keywords:** ozone, hydrogen peroxide, stable isotope analysis, oxygen isotope, dissolved organic matter, olefins, Suwannee river fulvic acid, Suwannee river humic acid, SRFA, SRHA

## Abstract

Reactivity-related characterization of dissolved organic matter (DOM) is crucial for mitigating undesirable byproduct formation during ozonation. However, the characterization of DOM remains challenging due to its molecular complexity, especially in distinguishing olefin- and phenol-type moieties, primary O_3_ reactive sites responsible for generating carbonyl-containing byproducts. Here, we present a novel approach based on stable oxygen isotope analysis of H_2_O_2_, a common byproduct of ozone reactions, to differentiate between these two moieties. The natural abundance oxygen isotopic signatures of residual H_2_O_2_ (δ^18^O_H_2_O_2__) after ozonation with 13 model compounds (6 olefins and 7 phenols) at pH 3 and 7, and their pH-dependency, Δ^18^O_H_2_O_2__ (δ^18^O_H_2_O_2__ (pH 7) – δ^18^O_H_2_O_2__ (pH 3)), enabled the distinction of olefins and phenols. Olefins exhibited near-zero or positive Δ^18^O_H_2_O_2__ (0.4–9.0‰), whereas phenols showed negative Δ^18^O_H_2_O_2__ (−14.2 – −1.6‰). These contrasting trends allowed derivation of an empirical correlation linking Δ^18^O_H_2_O_2__ to olefinic-phenolic fractions in mixtures, resulting in estimation of molar fractions of the two moieties in DOM isolates. This stable-isotope based approach offers unique mechanistic insights into H_2_O_2_ formation mechanisms for widespread applications in H_2_O_2_-generating reactions, as well as for synergistic use with existing DOM characterization techniques.

## Graphical abstract

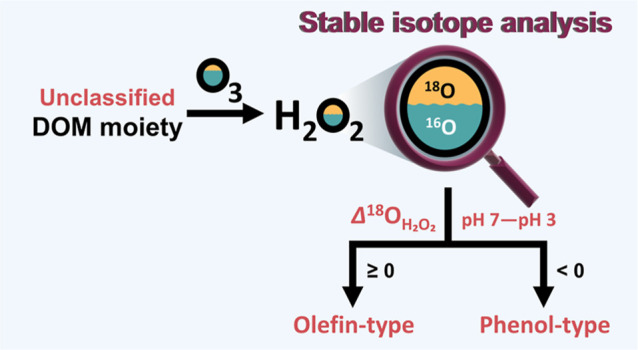

## 1 Introduction

Ozonation has been applied for more than a century for water disinfection and its use has increased in recent years owing to concerns with aquatic micropollutants and the rising need for water reuse.^1^ However, micropollutant abatement is limited by dissolved organic matter (DOM) because DOM consumes the major fraction of ozone, leading to the formation of oxidation/disinfection byproducts (DBPs).^2–5^ Thus, understanding the structural composition of DOM and its reactivity is crucial for assessing the treatment performance and mitigation of the formation of undesired DBPs. A key challenge in this endeavor is the complexity of DOM’s molecular structure and its characterization with regard to reactions with ozone.^6^

In fact, phenolic and olefinic groups are key sites for disinfection/oxidation processes and they exhibit high second-order rate constants for their reactions with ozone^1,5,7^ Phenolic moieties, which are among the dominant moieties in DOM, contribute significantly to ozone consumption due to their electron-rich character.^4,8–10^ Olefinic moieties are present at lower concentrations and react with ozone via cycloaddition reactions at the C=C double bond, with distinct transformation pathways.^1,7,11–13^ Despite their different reaction mechanisms with ozone, both phenolic and olefinic moieties are precursors for the formation of aldehydes and ketones, which are a major class of ozonation DBPs.^14^ For this reason, DBP formation by the two types of precursors is hardly distinguishable in complex water matrices.^11,14^ However, the characterization of the two types of ozone-reactive moieties is important in water treatment as it influences the efficiency of ozone utilization and the extent of DBP formation.^11^ Overall, this affects both treatment performance and regulatory compliance.

Current efforts on the characterization and quantification of phenolic moieties in DOM^15^ include techniques such as indirect titration with calcium acetate and barium hydroxide,^16^ potentiometric titration,^17^ oxidative titration with ClO_2_,^4^ spectrophotometry with the Folin–Ciocalteu phenol reagent,^18,19^ gas chromatography (GC) or high-performance liquid chromatography (HPLC) following copper oxide oxidation,^20,21^ ^13^C/^29^Si-nuclear magnetic resonance (NMR) spectroscopy,^22^ and electron-donating capacity (EDC) measurements.^23^ By contrast, the quantification of olefinic moieties remains largely unexplored. Due to the lack of selective reagents for olefins, studies have so far relied on direct detection of double bonds by ^13^C NMR and Fourier Transform Infrared (FT-IR) spectroscopy.^24,25^ However, these techniques cannot distinguish olefinic moieties from other unsaturated functional groups and do not provide quantitative insights due to spectral overlap and low sensitivity in complex DOM matrices.

Recently, compound-specific isotope analysis (CSIA) has been applied as a complementary approach for identifying reaction pathways and precursors of DBPs in water treatment. With this application of CSIA, changes in natural abundance stable isotope ratios of reaction products, including DBPs, have been evaluated for selected precursor compounds.^26^ For example, CSIA of δ^13^C and δ^15^N in nitrosamines formed during chloramination has been utilized to trace nitrogen-containing precursors in the water matrix.^27,28^ In another study δ^13^C analysis of chloroform has been applied to distinguish between chlorine reactions with resorcinol- and phenol-type precursors.^29,30^ A CSIA-based approach has also been developed to distinguish olefinic and phenolic groups through stable oxygen isotope analysis of hydrogen peroxide (as δ^18^O of H_2_O_2,_ δ^18^O_H_2_O_2__), a common ozonation byproduct formed from both functional groups.^11^ A novel analytical method has been developed for determining δ^18^O of H_2_O_2_ (δ^18^O_H_2_O_2__) from ozonated samples, and the pH-dependence of δ^18^O_H_2_O_2__ after complete ozone consumption by phenol and olefins was investigated. It demonstrated a different characteristic pH-dependence for phenol and olefins, suggesting that δ^18^O_H_2_O_2__, particularly its pH-dependent behavior, could serve as a potential indicator to differentiate these two functional groups.^11^

This study aims to establish a method for distinguishing phenolic and olefinic moieties in DOM, by systematically investigating the pH-dependent δ^18^O_H_2_O_2__ for a broad range of model compounds. To that end, the δ^18^O_H_2_O_2__ trends for six model olefins and seven substituted phenols were elucidated, along with the underlying mechanisms of H_2_O_2_ formation. On that basis, an empirical correlation equation between Δ^18^O_H_2_O_2__ and the relative proportion between olefins and phenols was established. Finally, this equation was applied to DOM isolates to quantify their olefinic and phenolic fractions.

## 2 Methods

### 2.1 Materials and Reagents

All information related to chemicals, DOM isolates, and the ozone stock solution used in this study is provided in Text S1 in the Supporting Information.

### 2.2 Choice of Model Compounds

A set of model compounds was chosen to represent olefinic and phenolic moieties in DOM and beyond (Table 1). The selected compounds feature simple structures, diverse functional groups with well-known ozone mechanisms to determine δ^18^O_H_2_O_2__. Six olefinic model compounds including an unsaturated alcohol (3-buten-2-ol), unsaturated acids (*trans*-cinnamic acid, acrylic acid, and methacrylic acid), and a chlorinated olefin (*trans*-dichloroethene) and seven phenolic compounds with a wide range of electron-donating/withdrawing substituents in para position were selected (hydrogen, methyl, methoxy, hydroxyl, nitro, and chloro groups) due to a significant influence on product formation.^9^

**Table 1:**
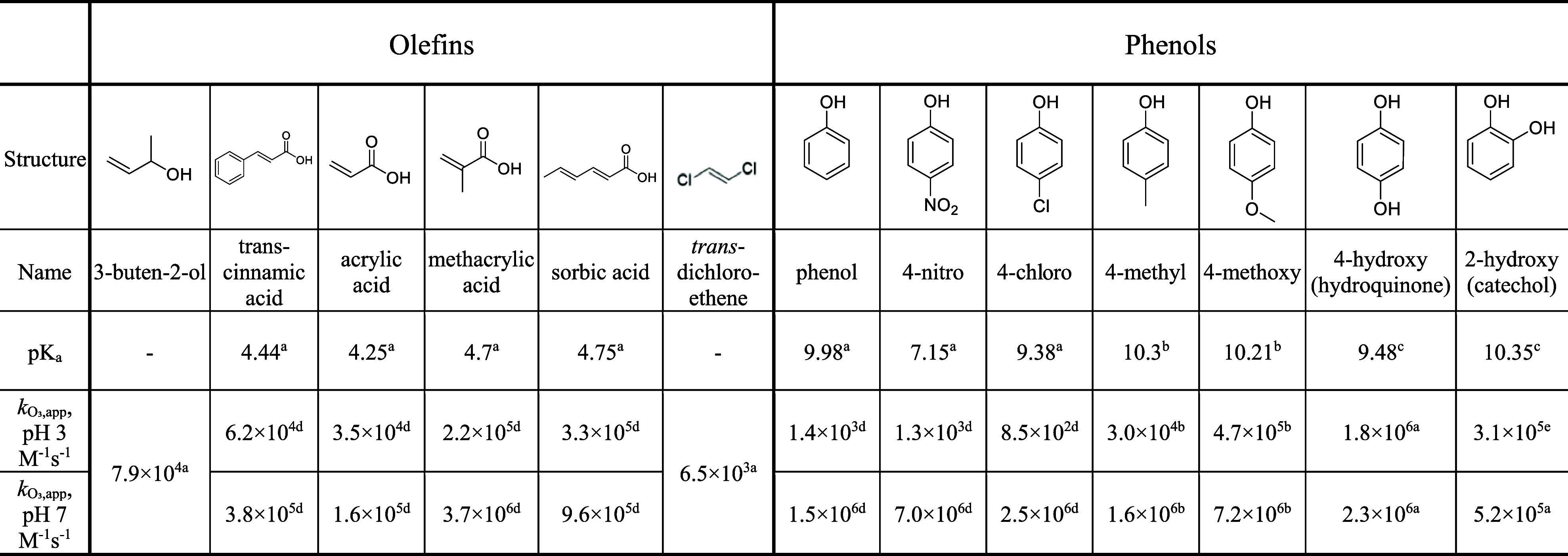
Selected Olefinic and Phenolic Model Compounds and the Corresponding p*K*_a_ Values and the Apparent Second-Order Rate Constants for the Reactions with Ozone at pH 3 and 7^a^

a *k*_O3,app_ denotes the apparent second-order rate constants for the reaction with ozone at a specific pH, expressed in units of M^–1^ s^–1^. von Sonntag and von Gunten.^7^

b Tentscher et al.^9^

c Shen et al.^59^ Values calculated based on the known species-specific second-order rate constants in Table S1 and the respective p*K*_a_ ^11^

e Gurol et al.^60^

### 2.3 Ozonation Conditions

Ozonation experiments were conducted by spiking an ozone stock solution (1.6–1.9 mM) into 100 mL of reaction solutions.^11^ Experimental conditions for ozonation of model compounds are presented in Table S1 and were designed to ensure selective analysis of H_2_O_2_ formation from the O_3_-compound reactions while minimizing potential interference by secondary reactions. To achieve this, a molar excess of the model compound relative to the O_3_ dose ([model compound]/[O_3_] ≥ 2) was applied, along with 33 mM of dimethyl sulfoxide (DMSO) to effectively scavenge hydroxyl radicals (^•^OH). DMSO was chosen as a scavenger, because it leads to minimal production of H_2_O_2_ (<1%) compared to tertiary butanol (25–30%).^11,31,32^ Model compound concentrations and O_3_ doses were determined based on the apparent second-order rate constants (Tables 1 and S1) to ensure 95% O_3_ consumption by the compound and 95% ^•^OH scavenging by DMSO.^7^ Two pH levels, 3 and 7, with 10 mM phosphate buffer, were employed to examine the pH-dependence of H_2_O_2_ formation.^9,11^ For ozonation of cinnamic acid-phenol mixtures and DOM isolates, the same conditions are used for the model compounds except for the compound concentrations and O_3_ doses. Mixtures contained 300 μM total compounds with molar [compounds]/[O_3_] ratios from 0.3 to 1.5. DOM isolates were treated with O_3_ doses of 5 and 7 mmol_O3_ gC^–1^ at 45 mg C L^–1^.

### 2.4 Analytic Methods

#### 2.4.1 H_2_O_2_ Quantification

H_2_O_2_ concentrations after ozonation were measured using a singlet oxygen (^1^O_2_) phosphorescence method^9,11^ as the primary technique and the Allen’s reagent method^33^ as a complementary approach. The ^1^O_2_ phosphorescence method was used for all ozonation samples due to its high selectivity and precision in complex aqueous matrices.^11^ In this method, 1 mL of chlorine reagent (50 mM in 100 mM phosphate buffer, pH 7.0) was added to 1 mL of sample, generating ^1^O_2_ via the reaction between HO_2_^–^ and HOCl.^9,11,34^ The resulting ^1^O_2_ phosphorescence emission was detected at 1270 nm using a near-infrared photomultiplier tube (NIR-PMT). The Allen’s reagent method was used exclusively for olefinic samples to quantify organic peroxides, as phenolic transformation products were found to interfere with the reagent.^9^ This method includes oxidation of iodide in the Allen’s reagent (400 mM KI, 50 mM NaOH, 0.16 mM (NH_4_)_6_Mo_7_O_24_·4H_2_O) by H_2_O_2_ and organic peroxides, producing triiodide (I_3_^–^), which was quantified after 1 min (for H_2_O_2_) and 20 min (for H_2_O_2_ + organic peroxides) using a spectrophotometer at 351 nm (ε_I_3_^–^_ = 25,700 M^–1^ cm^–1^, Cary 1, Varian).^11,35^ The detailed procedures and limits of quantification (LOQ) have been described in a previous study.^11^

#### 2.4.2 δ^18^O Measurements

The procedure for a δ^18^O_H_2_O_2__ determination has been described in a previous study.^11^ The detailed procedure applied here is also provided in Text S2. Briefly, three steps were carried out: (1) sample pretreatment: samples were acidified to pH 3 using phosphoric acid to stabilize H_2_O_2_ and purged with N_2_ gas to remove dissolved oxygen. (2) Conversion of H_2_O_2_ to O_2_: H_2_O_2_ in the sample was converted to O_2_ at pH 7 by adding NaOCl with NaOH, followed by chlorine quenching with ascorbic acid. (3) ^18^O/^16^O ratio (^18^ *R*) measurement: the ^18^ *R* of the formed gaseous dissolved O_2_ was determined using gas chromatography-isotope ratio mass spectrometry (GC/IRMS, Thermo Fisher Scientific) consisting of a GC coupled via a Conflo IV interface to a Delta V Plus isotope ratio mass spectrometer. To improve the precision compared to a previous study,^11^ a high and identical NaOCl concentration (34 mM) was added for the conversion of H_2_O_2_ to O_2_ for all experiments, ensuring an equally high conversion efficiency. Additionally, a highly acidic ascorbic acid quencher (pH 0.8, adjusted with phosphoric acid) was introduced to lower the final sample pH to <3, preventing O_2_ consumption by ascorbic acid.^36^ As a result, the detection limit was enhanced to 10 μM H_2_O_2_ with commercial H_2_O_2_ solutions, and the O_2_ conversion efficiency from H_2_O_2_ improved from the previous (90 ± 10)%^11^ to (94 ± 5)% (Figure S1), yielding high conversions of (94 ± 8)% for model compounds (Table S2). This leads to a significantly enhanced precision of δ^18^O_H_2_O_2__ measurements for experimental replicates.

δ^18^O was calculated based on the relative abundance of the isotopic ratio compared to a reference O_2_ gas (99.995%) used as an internal reference.^26^

1 δ18Osample=(Rsample18/Rreference18)−1

where δ^18^O_sample_ is the oxygen isotopic signature of the sample, ^18^ *R*_sample_ and ^18^ *R*_reference_ are the ^18^O/^16^O ratios in the sample and the reference, respectively. δ^18^O_H_2_O_2__ was determined with blank corrections to eliminate background contributions from the experimental matrix^37,38^

2 $$\delta^{18}O_{H_{2}O_{2}}=\frac{\delta^{18}O_{H_{2}O_{2},sample}\times {\;A}_{H_{2}O_{2},sample}-\delta^{18}O_{blank}\times A_{blank}}{A_{H_{2}O_{2},sample}-A_{blank}}$$

where δ^18^O_H_2_O_2_,sample_ and *A*_H_2_O_2_,sample_ represent the oxygen isotopic signature and peak area of mass 32 of H_2_O_2_ in the H_2_O_2_-containing sample, while δ^18^O_blank_ and *A*_blank_ correspond to values obtained from blanks, containing the same matrix as the sample but without ozonation, analyzed with the same analytical sequence. To quantify the pH dependence in δ^18^O_H_2_O_2__, Δ^18^O_H_2_O_2__ was calculated as follows

$$\Delta^{18}O_{H_{2}O_{2}}=\delta^{18}O_{H_{2}O_{2}}\left(pH7\right)-\delta^{18}O_{H_{2}O_{2}}\left(pH3\right)$$ 3

#### 2.4.3 Compound and DOC Quantification

To determine cinnamic acid and phenol consumption during ozonation, benzaldehyde (formed with a 1:1 stoichiometry from cinnamic acid ozonolysis)^39^ and phenol were quantified using high-performance liquid chromatography coupled to a diode array detector (HPLC-DAD, Ultimate 3000, Thermo Scientific). The measurement conditions are detailed in Text S3. The dissolved organic carbon (DOC) concentration of DOM isolates was determined using a total organic carbon analyzer (TOC-VCPH, Shimadzu).

### 2.5 δ^18^O_H_2_O_2__ of Commercial H_2_O_2_

To clarify the δ^18^O_H_2_O_2__ evolution in O_3_-model compound reactions, commercial H_2_O_2_ solution (30% w/w, Sigma-Aldrich) was reacted with formaldehyde, formic acid, *trans*-dichloroethene, O_3_, and DOM isolates, and the δ^18^O_H_2_O_2__ of residual H_2_O_2_ was measured. The reaction conditions are provided in Figure S5.

## 3 Results and Discussion

### 3.1 H_2_O_2_ Yields of Model Compounds

Before investigating the isotopic properties of the H_2_O_2_, the H_2_O_2_ formed from the ozonation of the selected model compounds at pH 3 and 7 was quantified as % molar yield based on the O_3_ dose (Figure 1a). The selected model compounds had variable H_2_O_2_ yields dependent on the H_2_O_2_ formation mechanisms. Olefins, except *trans*-dichloroethene, exhibited higher H_2_O_2_ yields (37–96%) than phenols (1–30%). This is because ozonation of olefins forms H_2_O_2_ via the Criegee mechanism where O_3_ cleaves an alkene bond, stoichiometrically generating peroxides (organic peroxide + H_2_O_2_) and carbonyl products with a 100% molar yield (Figure 2a).^7^ Therefore, for most olefins, the equilibrium reaction between organic peroxides and H_2_O_2_ (**3** ⇄ **4** + H_2_O_2_, Figure 2) primarily determines the H_2_O_2_ yield.^11^ In contrast, phenols consume O_3_ by diverse reactions including the Criegee mechanism, but also oxygen addition with ^1^O_2_ generation and electron transfer with ^•^OH formation.^7^ Therefore, phenol ozonation is accompanied by relatively lower H_2_O_2_ yields.^8,9,40^ Details of H_2_O_2_ yields will be discussed along with its formation mechanisms in the following section.

**Figure 1:**
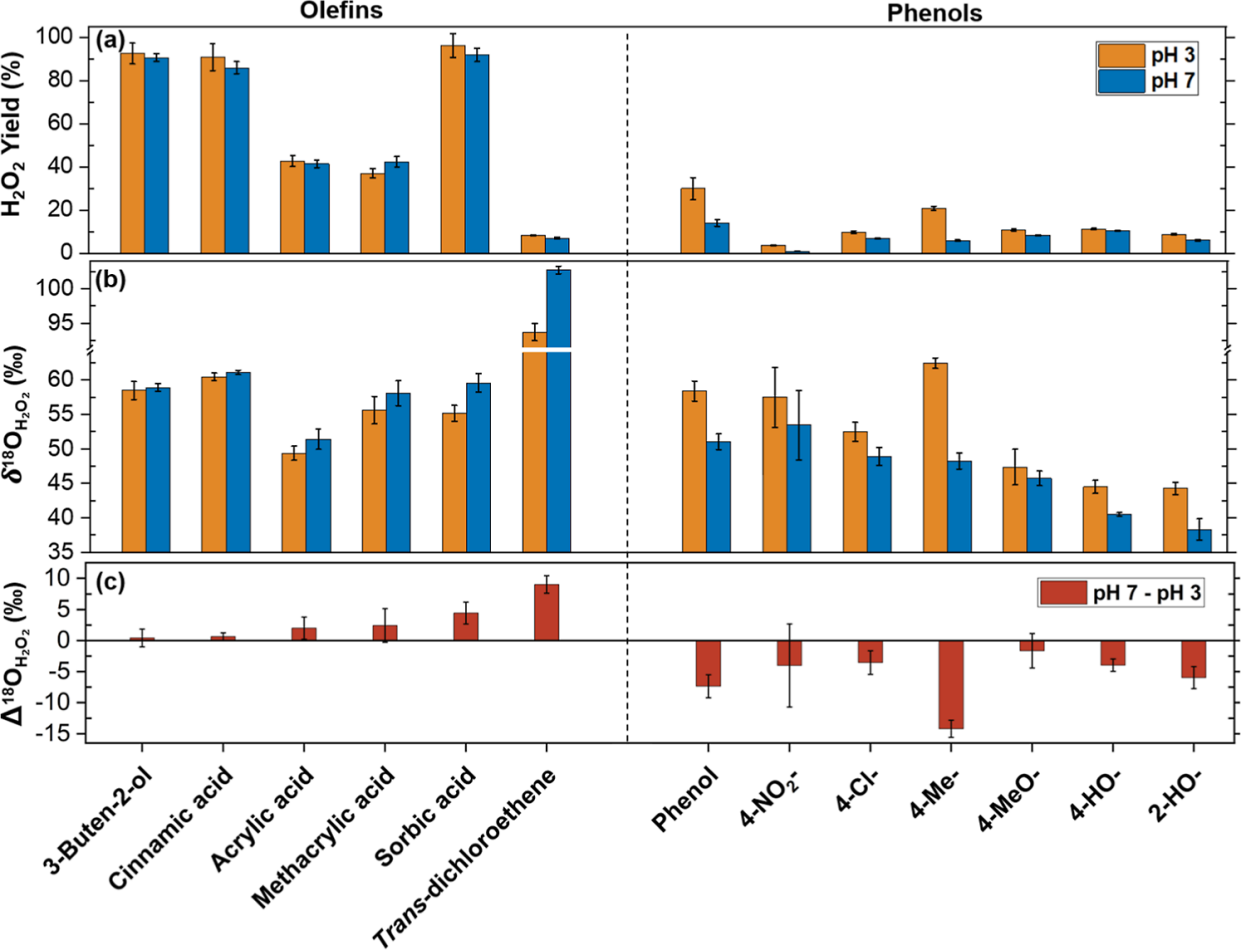
Reactions of ozone with olefinic and phenolic compounds. (a) H_2_O_2_ yields (% relative to consumed O_3_), (b) δ^18^O_H_2_O_2__, and (c) Δ^18^O_H_2_O_2__ (δ^18^O_H_2_O_2__ (pH 7) – δ^18^O_H_2_O_2__ (pH 3)) for the selected model compounds. [Phosphate buffer] = 10 mM (pH 3 or 7), [DMSO] = 33 mM, molar [Compound]/[O_3_] = 2–5. Detailed experimental conditions and number of replicates for each compound are provided in Tables S1 and S2, respectively.

**Figure 2:**
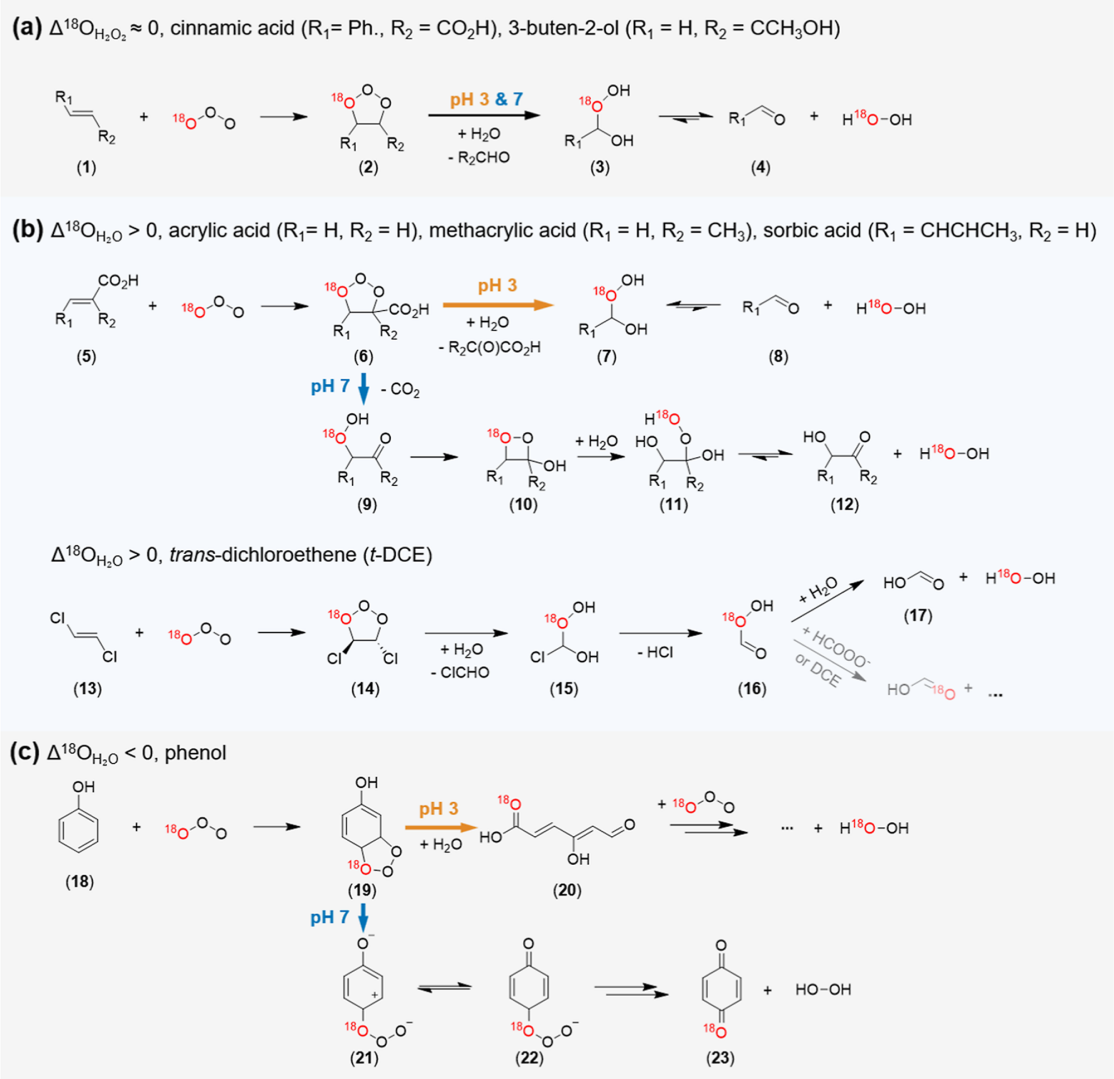
Proposed H_2_O_2_ formation mechanisms as a function of pH during ozonation of olefinic and phenolic model compounds categorized based on Δ^18^O_H_2_O_2__. (a) Category I olefins (Δ^18^O_H_2_O_2__ ≈ 0), (b) category II olefins (Δ^18^O_H_2_O_2__ > 0), and (c) category III phenol (Δ^18^O_H_2_O_2__ < 0). The black O atoms represent ^16^O, while the red O atoms indicate ^18^O.

### 3.2 δ^18^O_H_2_O_2__ of Model Compounds

Figure 1b shows δ^18^O_H_2_O_2__, which is observed after ozonation of the model compounds at pH 3 and 7, indicative of the footprint of ^18^O in O_3_ and the ensuing reactions leading to H_2_O_2_. Variations in δ^18^O_H_2_O_2__ for different pH values suggest that the ozonated compound has a pH-dependent H_2_O_2_ formation pathway. To quantify the pH-driven difference and evaluate the trend across compounds, Δ^18^O_H_2_O_2__ was calculated by Δ^18^O_H_2_O_2__ = δ^18^O_H_2_O_2__ (pH 7) – δ^18^O_H_2_O_2__ (pH 3) and is depicted in Figure 1c.

The Δ^18^O_H_2_O_2__ values for model compounds disclose a clear contrast between olefins and phenols, with near-zero or positive Δ^18^O_H_2_O_2__ for olefins and negative Δ^18^O_H_2_O_2__ for phenols. This stark difference highlights the potential of Δ^18^O_H_2_O_2__ as an indicator for probing for olefinic and phenolic moieties in complex organic matrices. For a better understanding of the Δ^18^O_H_2_O_2__ trends, we categorized the model compounds into three groups in the ensuing discussion: (I) Δ^18^O_H_2_O_2__ ≈ 0, including 3-buten-2-ol and cinnamic acid, (II) Δ^18^O_H_2_O_2__ > 0, including acrylic acid, methacrylic acid, sorbic acid, and *trans*-dichloroethene, and (III) Δ^18^O_H_2_O_2__ < 0, including all phenolic compounds. The underlying mechanisms driving the Δ^18^O_H_2_O_2__ trends for each category are illustrated in Figure 2.

#### 3.2.1 Category I: Δ^18^O_H_2_O_2__ ≈ 0 (3-Buten-2-ol and Cinnamic Acid)

The category I compounds, 3-buten-2-ol and cinnamic acid, exhibited near-zero Δ^18^O_H_2_O_2__ values (0.4 ± 1.4‰ and 0.6 ± 0.6‰, respectively), indicating no pH-dependence in their H_2_O_2_ formation mechanism, as illustrated in Figure 2a. Regardless of pH, category I compounds showed ∼100% H_2_O_2_ yields and ∼60‰ δ^18^O_H_2_O_2__ (Figure 1a,b, and Table S2) through the Criegee mechanism. The category I mechanism has been well-documented in a previous study as a baseline case.^11^ Briefly, during the Criegee mechanism, ^18^O in O_3_ preferably accumulates in H_2_O_2_ rather than in the carbonyl product (R_2_CHO) for a ∼100% conversion of O_3_ to H_2_O_2_. This leads to a significant enrichment of δ^18^O_H_2_O_2__ up to ∼60‰ relative to the δ^18^O in O_3_ (28‰, see Text S4 and Figure S2 for details regarding δ^18^O of O_3_). This O isotope fractionation originates from preferential bond cleavage of ^16^O–^16^O compared to ^16^O–^18^O in the Criegee ozonide (**2**, Figure 2a) due to a higher bond dissociation energy for bonds containing heavier isotopes. This behavior corresponds to a normal kinetic isotope effect (KIE > 1).^41^ If O_3_ includes ^18^O at the edge site (^18^O^16^O^16^O), as depicted in Figure 2a, the weaker bond (^16^O–^16^O) in the ozonide (**2**, Figure 2a) is cleaved to generate ^18^O^16^OH in the α-hydroxyalkylhydroperoxide (**3**, Figure 2a), leading to ^18^O in H_2_O_2_. For the other isotopomer ^16^O^18^O^16^O, ^18^O is always incorporated into H_2_O_2_ (Figure S3).

While 3-buten-2-ol exhibited ∼100% H_2_O_2_ yield in the ^1^O_2_ method (Figure 1a), it exhibited organic peroxide formation in the Allen’s method (58% at pH 3 and 47% at pH 7, Figure S4 and Table S2). This discrepancy likely arises from the chlorination process in the ^1^O_2_ method, where chlorine converts both organic peroxides and H_2_O_2_ to O_2_. As the δ^18^O_H_2_O_2__ analyses also involve a chlorination process for the H_2_O_2_-to-O_2_ conversion, the organic peroxides were similarly converted to O_2_, resulting in the same level of ^18^O accumulation as observed for cinnamic acid (∼60‰ δ^18^O_H_2_O_2__ at both pH levels, Figure 1b). In summary, category I compounds exhibit pH-independent ^18^O-enriched H_2_O_2_ formation, resulting in Δ^18^O_H_2_O_2__ ≈ 0.

#### 3.2.2 Category II: Δ^18^O_H_2_O_2__ > 0 (Acrylic Acid, Methacrylic Acid, Sorbic Acid, and *Trans*-Dichloroethene)

Category II includes most acids (acrylic acid, methacrylic acid, and sorbic acid) and *trans*-dichloroethene, which showed positive Δ^18^O_H_2_O_2__ values (2.0 ± 1.8, 2.4 ± 2.7, 4.4 ± 1.8, and 9.0 ± 1.4, respectively, Figure 1c) and their potential H_2_O_2_ formation mechanism is illustrated in Figure 2b. While these olefins undergo a Criegee mechanism, comparable to category I compounds, they demonstrate δ^18^O_H_2_O_2__ values lower than 60‰ (Figure 1b and Table S2), except *trans*-dichloroethene. This decrease in δ^18^O_H_2_O_2__ is likely associated with the formation of organic peroxides (α-hydroxyalkylhydroperoxides, **7** and **11**, Figure 2b), which coexist in equilibrium with H_2_O_2_.^11^ Application of the Allen’s method disclosed organic peroxides yields for acrylic acid, methacrylic acid, and sorbic acid (Figure S4 and Table S2). Figure 3b illustrates a negative trend for δ^18^O_H_2_O_2__ as a function of the organic peroxide formation (as % of total peroxides) for olefinic model compounds. This result implies that organic peroxide formation lowers the δ^18^O_H_2_O_2__ because the equilibrium between organic peroxide and H_2_O_2_ depletes ^18^O in H_2_O_2_.

**Figure 3:**
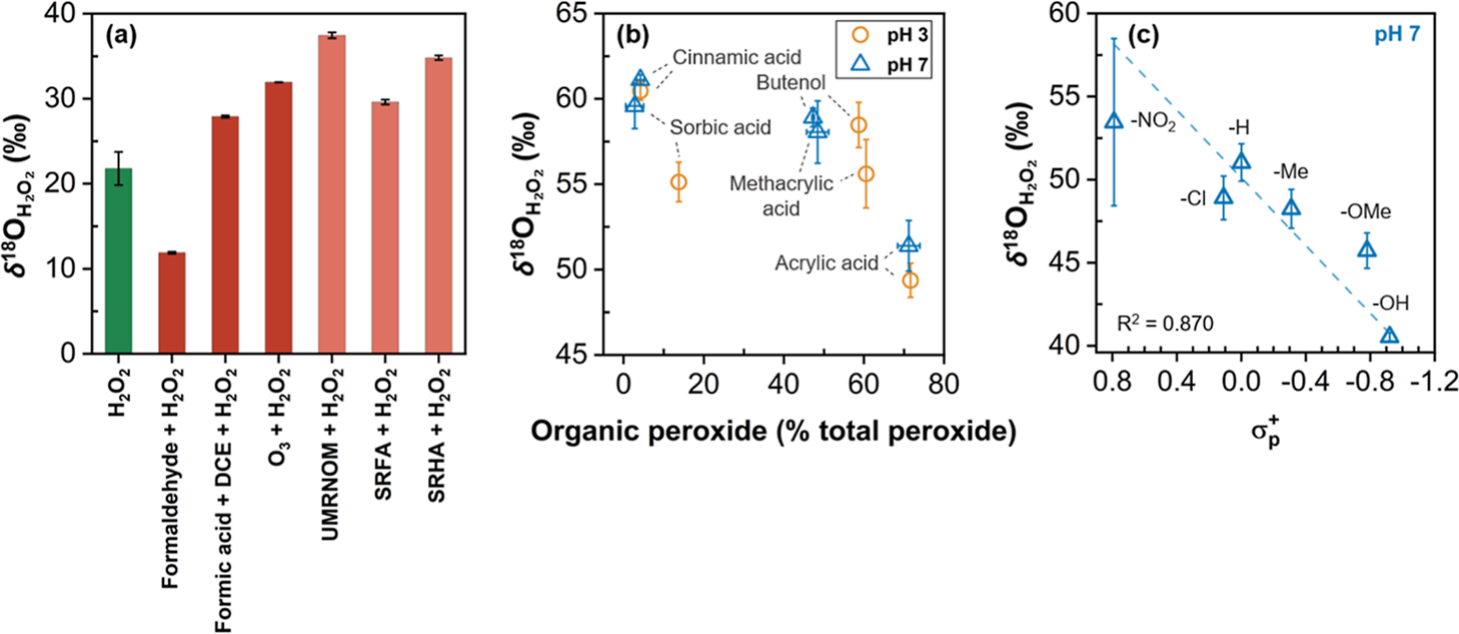
Oxygen isotope patterns of commercial H_2_O_2_ or the formed H_2_O_2_ from ozonated model compounds. δ^18^O_H_2_O_2__ for (a) commercial H_2_O_2_ after reactions with various compounds, (b) ozonation of olefins (as a function of organic peroxide yield), and (c) ozonation of para-substituted phenols as a function of the Hammett constants. Experimental details for (a) are provided in Figure S5. The δ^18^O_H_2_O_2__ values shown in (b,c) are taken from Figure 1b and rearranged to illustrate the trends.

To explore the O isotope fractionation in this equilibrium reaction, the equilibrium reaction of H_2_O_2_ and formaldehyde with an organic peroxide (hydroxymethylhydroperoxide) (eq 4) was investigated.

4 $$CH_{2}O+{\;H}_{2}O_{2}\;⇌\;HOCH_{2}OOH$$

After 62% of H_2_O_2_ was converted to hydroxymethylhydroperoxide in equilibrium (Figure S5a), the δ^18^O_H_2_O_2__ of the residual H_2_O_2_ was determined to be 11.9‰, which is significantly lower than δ^18^O_H_2_O_2__ of the commercial H_2_O_2_ (δ^18^O_H_2_O_2__ = 20.2‰) (Figure 3a). This indicates that ^18^O is preferentially bound to the organic peroxide rather than H_2_O_2_, which is consistent with the hypothesized higher bond strength of C–^18^O bond compared to a C–^16^O bond.^41^ As a result, the equilibrium reaction of **7** ⇌ **8** + H_2_O_2_ or **11** ⇌ **12** + H_2_O_2_ lowers δ^18^O_H_2_O_2__ for category II olefins (Figure 2b).

The pH-dependence of the δ^18^O_H_2_O_2__ arises from pH-induced changes in the mechanisms of category II compounds (Figure 2b).^11,42^ At lower pH, the Criegee ozonide (**6**, Figure 2b) undergoes bond breakage between α- and β-carbons of the acid group, leading to an α-ketoacid and hydroxyalkylhydroperoxides (**7**). In contrast, at higher pH, the deprotonated carboxyl group undergoes decarboxylation, forming first an organic peroxide (**9**), which is then transformed into dioxetane (**10**) and subsequently hydrolyzes to a second organic peroxide (**11**). Organic peroxides induced by decarboxylation eventually have higher molecular weights (longer alkyl chains) (**11**) compared to the products formed at lower pH (**7**). Longer alkyl chains, with stronger tendencies to act as electron donors, promote the decomposition of an organic peroxide by stabilizing the ensuing carbonyl compound.^43^ For instance, in the case of acrylic acid,^42^ glycolaldehyde, a longer-chain transformation product from the higher pH route, has a much lower equilibrium constant for the organic peroxide formation (*K* = 43.3 M^–1^, *K* = [organic peroxide]_eq_/([H_2_O_2_]_eq_ × [Carbonyl]_eq_)) than formaldehyde (*K* = 164 M^–1^),^43^ a shorter-chain organic peroxide from the lower pH route. Consequently, at pH 7, lower organic peroxide concentrations are formed (higher δ^18^O_H_2_O_2__) than at pH 3, leading to a positive Δ^18^O_H_2_O_2__.

*Trans*-dichloroethene also showed positive Δ^18^O_H_2_O_2__ values but it follows a distinct ozonation mechanism ascribed to the presence of a Cl atom (Figure 2b).^12^ Chlorinated organic peroxide (**15**, Figure 2b), derived from Criegee ozonide (**14**), undergoes dehydrochlorination, yielding performic acid (**16**). Performic acid (**16**) can follow two pathways: hydrolysis to H_2_O_2_ (upper pathway) or self-decomposition and reaction with *trans*-dichloroethene (lower pathway). Given the relatively low H_2_O_2_ yields (7–8%, Figure 1a), the lower pathway appears to dominate. In the lower pathway, the reactive performic acid (**16**) reacts with HCOOO^–^ (self-decomposition) or *trans*-dichloroethene.^44,45^ These reactions involve a bond cleavage of O–O in the hydroperoxide group, leading to an O isotope fractionation through a normal KIE, as discussed earlier. Therefore, the performic acid containing a light oxygen bond (^16^O–^16^O) preferentially decomposes (lower pathway), while the ^18^O-containing-performic acid (**16**) preferably hydrolyzes to H_2_O_2_ that preserves the O–O bond (upper pathway). This offers a plausible explanation for the notably high δ^18^O_H_2_O_2__ values observed from *trans*-dichloroethene ozonation (93.7‰ at pH 3 and 103‰ at pH 7).

The ^18^O enrichment in H_2_O_2_ through the self-decomposition and reaction with *trans*-dichloroethene was confirmed in an experiment with H_2_O_2_ and formic acid. H_2_O_2_ showed increased δ^18^O_H_2_O_2__ after a 6 h reaction with formic acid and *trans*-dichloroethene (Figure 3a), while the total peroxide concentration (H_2_O_2_ + performic acid) decreased (further details in Figure S5b). The lower H_2_O_2_ yield and higher δ^18^O_H_2_O_2__ at pH 7 than at pH 3 can be attributed to an increased reactivity of performic acid at higher pH levels,^45^ resulting in a positive Δ^18^O_H_2_O_2__. In conclusion, category II compounds exhibited positive Δ^18^O_H_2_O_2__ values, driven by pH-dependent O isotope fractionation arising from equilibrium reactions of H_2_O_2_ with aldehydes or decomposition of organic peroxides.

#### 3.2.3 Category III: Δ^18^O_H_2_O_2__ < 0 (Phenolic Compounds)

All phenolic model compounds are classified as category III, showing negative Δ^18^O_H_2_O_2__ values (Figure 1c) and H_2_O_2_ formation mechanisms of phenol are presented in Figure 2c. The ozonolysis of phenols includes diverse pathways and transformation products.^8,9,11,40^ Here, the focus is on mechanisms contributing to H_2_O_2_ formation. H_2_O_2_ formation from phenol can be split into an upper pathway in Figure 2c (**19** → **20**), followed by Criegee-type H_2_O_2_ formation, and a lower pathway in Figure 2c (**19** → **21**), followed by H_2_O_2_ formation from the noncyclic ozonide (**21** and **22**). The upper pathway dominates at pH 3, where bidentate attack (**19**) forms a ring-opening product (**20**) and enables the Criegee reactions of the ensuing olefins with O_3_, to produce a higher H_2_O_2_ yield and δ^18^O_H_2_O_2__ (29.8% and 58.4‰, respectively) compared to pH 7 (14.0% and 51.0‰) (Table S2).

At pH 7, the deprotonated hydroxyl group in the monodentate attack intermediate (**21**) stabilizes a positive charge developed in the ring of the ozone adduct via charge redistribution (**21 ⇌ 22**), promoting the lower pathway (Figure 2c).^7^ The noncyclic ozonide (**22**) releases H_2_O_2_ along with the production of *para*-benzoquinone (**23**). Since the ozonide can be decomposed into ^1^O_2_ and O_3_^–^ (^•^OH) as well,^7^ the H_2_O_2_ yield is about half (14.0%) compared to pH 3 (29.8%). Furthermore, ^18^O in ozone bound to the C_4_ position of phenol has a higher bond dissociation energy than ^16^O.^41^ Therefore, the ^18^O preferentially remains at the acyl group (**23**) rather than being released with H_2_O_2_. This causes less ^18^O enrichment in H_2_O_2_ with a lower δ^18^O_H_2_O_2__ of 51.0‰ at pH 7 compared to pH 3 (58.4‰), resulting in a negative Δ^18^O_H_2_O_2__ value (−7.4 ± 1.9‰).

The pH-dependent branching of the H_2_O_2_ formation mechanism is compatible across all substituted phenols, while the aromatic substitution alters the transformation products, such as benzoquinone, catechol, cyclodienone, and hydroquinone.^9^ For phenols, the electron-donor/acceptor capacity of the substituents affects δ^18^O_H_2_O_2__. As illustrated in Figure 3c, δ^18^O_H_2_O_2__ of phenols at pH 7 exhibited a negative correlation with substituents’ Hammett constants (σ_p_^+^), which quantify the para-substituent’s ability to donate/accept electrons to/from the aromatic ring.^7,46^ Electron-donating substituents can offer enhanced stabilization of the positive charge in the aromatic ring of intermediate **21** (Figure 2c),^7,47^ promoting the lower pathway leading to a decrease in δ^18^O_H_2_O_2__. However, the Hammett correlation becomes insignificant at pH 3 (Figure S6), presumably due to a stronger influence of p*K*_a_, which determines the fraction of phenol/phenolate. For example, methylphenol, an outlier in the correlation at pH 3 (Figure S6), has the highest p*K*_a_ (Table 1) which leads to a low phenolate fraction and promotes the upper pathway of the H_2_O_2_ formation mechanism (Figure 2c). Hydroxyphenol isomers (hydroquinone and catechol) demonstrated similar H_2_O_2_ yields and δ^18^O_H_2_O_2__ values (Figure 1a,b, and Table S2), indicating coherent H_2_O_2_ formation mechanisms regardless of the substituent type and position. Overall, phenols consistently showed negative Δ^18^O_H_2_O_2__ values with a preferential non-Criegee-type H_2_O_2_ formation at pH 7, and the δ^18^O_H_2_O_2__ decreases proportionally to the electron-donating properties of the substituents.

### 3.3 Derivation of a Correlation Equation between Δ^18^O_H_2_O_2__ and the Fraction of Phenol (*f*_p_)

As a higher phenolic fraction (*f*_P_) in the matrix is hypothesized to yield more negative Δ^18^O_H_2_O_2__-values, we established an empirical correlation equation linking Δ^18^O_H_2_O_2__ values to the *f*_P_. This correlation was derived from mixtures of representative olefinic and phenolic compounds, namely cinnamic acid and phenol. The Δ^18^O_H_2_O_2__ of the cinnamic acid-phenol mixtures was measured at varying initial molar fractions (*f*_P_ = 0, 0.25, 0.5, 0.75, and 1) and specific ozone doses ([O_3_]/[total compounds] = 0.3, 0.75, 1.1, 1.5 mol mol^–1^). Figure 4a illustrates Δ^18^O_H_2_O_2__ as a function of varying specific ozone doses for different molar phenol fractions, while more detailed results, including δ^18^O_H_2_O_2__, H_2_O_2_ yields, and cinnamic acid/phenol consumptions at pH 3 and 7, are shown in Figure S7. As shown in Figure 4a, Δ^18^O_H_2_O_2__ became more negative with increasing phenol fraction. The lowered Δ^18^O_H_2_O_2__ with higher *f*_P_ is attributed to the decreased δ^18^O_H_2_O_2__ at pH 7 based on the increase of the fraction of the phenol-derived H_2_O_2_ (Figure S7a,c). Although Δ^18^O_H_2_O_2__ for mixture samples (*f*_P_ = 0.25–0.75) increased slightly by 2–3‰ as the ozone dose increased, leveling off at [O_3_]/[Compounds] > 1.1, the relative ordering of Δ^18^O_H_2_O_2__ with respect to *f*_P_ remained unchanged. The increase reflects an increased phenol contribution to H_2_O_2_ formation compared to cinnamic acid at higher O_3_ doses (Figure S7c). This consistency highlights the robustness of Δ^18^O_H_2_O_2__ as an indicator for mixtures of olefins and phenols, which could be applied for characterization of DOM.

**Figure 4:**
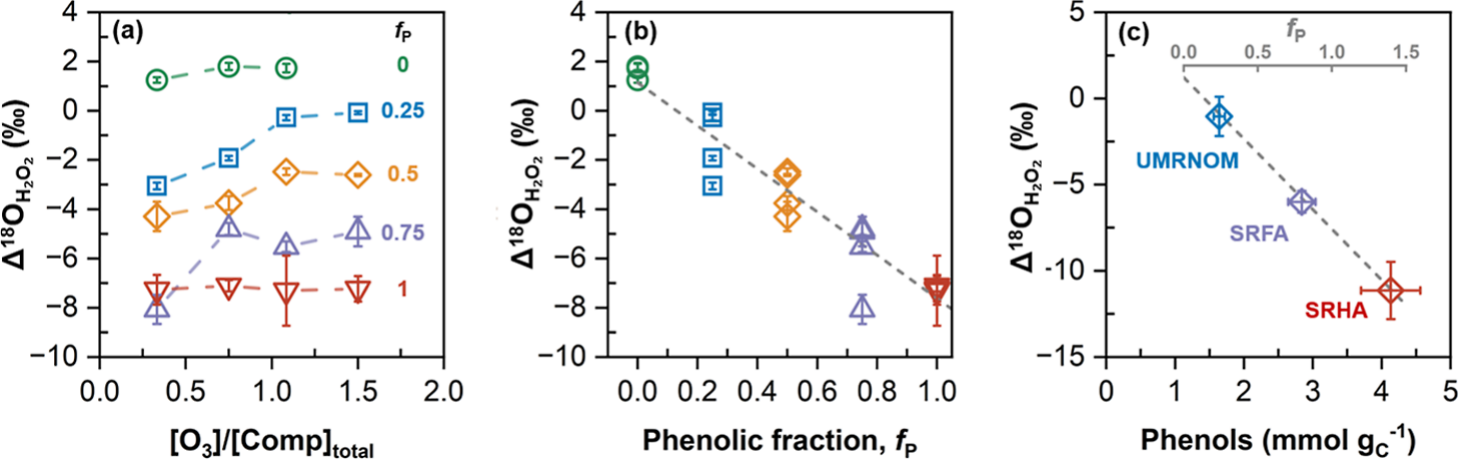
Ozonation of mixtures of olefinic and phenolic compounds and of DOM isolates. Δ^18^O_H_2_O_2__ for cinnamic acid-phenol mixtures as a function of (a) the specific O_3_ dose and (b) phenolic fraction (*f*_P_), with the gray dashed line representing a linear regression, which serves as an empirical Δ^18^O_H_2_O_2__-*f*_P_ correlation. (c) Δ^18^O_H_2_O_2__ for DOM isolates at an O_3_ dose of 7 mmol g_C_^–1^as a function of the molar phenolic content, determined by the ClO_2_ titration in a previous study,^4^ and *f*_P_, with the Δ^18^O_H_2_O_2__-*f*_P_ correlation depicted by the gray dashed line. [compounds]_total_ = 300 μM (a and b), [DOC] = 45 mg L^–1^ (c), [phosphate buffer] = 10 mM (pH 3 or pH 7), [DMSO] = 33 mM.

The Δ^18^O_H_2_O_2__ data plotted as a function of *f*_P_ in Figure 4b shows a linear correlation between Δ^18^O_H_2_O_2__ and the phenol fraction in the matrix. A linear regression enabled derivation of an empirical equation (eq 5), depicted by the gray dashed line.

$$\Delta^{18}O_{H_{2}O_{2}}=-8.8\times f_{P\;}+1.1$$ 5

This empirical correlation provides a tool to estimate molar phenols-olefins fractions in DOM from the measured Δ^18^O_H_2_O_2__. However, deriving a generally valid relationship for all DOM types is challenging due to the diverse composition of DOM and the correlation dependence on multiple factors, including compound-specific Δ^18^O_H_2_O_2__, H_2_O_2_ yields, and ozonation kinetics. Within the scope of this work, eq 5 serves as a proof of concept, while future studies should examine more complex phenol-olefin mixtures to assess how specific compounds affect such correlations.

In such experiments, the applied ozone doses should not exceed the ozone demand of the matrix. For example, a specific molar ozone dose ([O_3_]/[Compounds]) of 1.5 for the cinnamic acid solution (*f*_P_ = 0) at pH 7 left residual ozone in the solution, which consumes H_2_O_2_/HO_2_^–^,^48^ which eventually produces exceedingly high δ^18^O_H_2_O_2__ (116‰) and concomitant high Δ^18^O_H_2_O_2__ (51.5‰, Figure S8). In this case, the raised δ^18^O_H_2_O_2__ is a result of preferential consumption of light H_2_O_2_ by ozone with a normal KIE, which was corroborated by increased δ^18^O_H_2_O_2__ of commercial H_2_O_2_ during ozonation (Figure 3a; further details in Figure S5c). Such elevated Δ^18^O_H_2_O_2__ values can lead to significant misinterpretation, such as an underestimation of *f*_P_. This underlines the importance of determining the O_3_ demand of the matrix by an O_3_ titration,^4^ where incremental O_3_ doses are applied until a persistent residual O_3_ is observed. Performing the titration prior to Δ^18^O_H_2_O_2__ analysis ensures the O_3_ dose remains below the matrix O_3_ demand, preventing H_2_O_2_ consumption by residual O_3_.

### 3.4 Estimation of Phenolic and Olefinic Fractions in DOM Isolates

The δ^18^O_H_2_O_2__ analysis method and the Δ^18^O_H_2_O_2__-*f*_P_ correlation equation (eq 5) were applied in experiments with DOM isolates to estimate the olefinic and phenolic fractions. Three IHSS DOM isolates were selected based on their different phenolic compositions:^4,49^ Upper Mississippi river natural organic matter (UMRNOM), Suwannee river fulvic acid II (SRFA), and Suwannee river humic acid II (SRHA). Several quantification methods for phenolic moieties of these isolates have been widely applied, such as pH titration, EDC measurement, and ClO_2_ titration.^4,23,49^ However, the olefinic contents in these DOM samples remain largely unknown.

To explore the olefinic and phenolic fractions in the DOM isolates, their δ^18^O_H_2_O_2__ at pH 3 and 7 were determined (Figure S9b), along with H_2_O_2_ yields (Figure S9a). Specific O_3_ doses of 5 and 7 mmol_O3_ g_C_^–1^ were applied to DOM-containing solutions with DOC concentrations of 45 mg_C_ L^–1^. Both doses exhibited identical trends, indicating a minimal ozone dose impact. The following discussion focuses on 7 mmol_O3_ g_C_^–1^ because of its lower standard deviations. The resulting Δ^18^O_H_2_O_2__ values were −1.0 ± 1.2‰, −6.0 ± 0.6‰, and −11 ± 1.7‰ for UMRNOM, SRFA, and SRHA, respectively (Figure S9c).

To estimate molar fractions of olefinic and phenolic moieties, Δ^18^O_H_2_O_2__ was plotted as a function of the molar concentration of phenolic moieties (1.64 ± 0.08, 2.84 ± 0.20, and 4.13 ± 0.43 mmol_phenol_ g_C_^–1^ for UMRNOM, SRFA, and SRHA, respectively), determined by the ClO_2_ titration.^4^ As shown in Figure 4c, Δ^18^O_H_2_O_2__ displayed a good correlation with the phenol content of the DOM isolates. Using the measured Δ^18^O_H_2_O_2__ values in the derived Δ^18^O_H_2_O_2__-*f*_P_ correlation (dashed gray line, eq 5), *f*_P_ was 0.24, 0.82, and 1.0 (calculated as 1.4, but rounded by unity) for UMRNOM, SRFA, and SRHA, respectively. The corresponding olefinic fractions (1 – *f*_P_) are approximately 0.76, 0.18, and 0.00, giving estimated olefinic concentrations of 5.1, 0.64, and 0 mmol g_C_^–1^ for UMRNOM, SRFA, and SRHA, respectively. In agreement with studies on the DOM composition of SRFA, the olefinic content of SRFA was found to be low (18%) in the current study.^50^ The high phenolic contents of SRFA and SRHA may result from the resin-based isolation process collecting mostly aromatics.^49,51^ In particular, during SRHA isolation, the precipitation step retains more phenols with longer alkyl-chains than SRFA, likely masking olefinic precursors and introducing high uncertainty in their determination.^52^ In contrast, UMRNOM, isolated using reverse osmosis,^49^ appears to contain a significant fraction of olefinic moieties. However, this will have to be tested more rigorously in future studies.

It is worth mentioning that δ^18^O_H_2_O_2__ measured at a single pH level has some limitations for identifying precursor compounds. While Δ^18^O_H_2_O_2__ correlates well with the phenolic content, the δ^18^O_H_2_O_2__ at both pH levels showed no trend, with unexpected high values (60–90‰, Figure S9b), compared to those of model compounds (40–60‰, except *trans*-dichloroethene, Figure 1b). The higher δ^18^O_H_2_O_2__ values may result from H_2_O_2_ consumption by H_2_O_2_-reactive compounds in the DOM isolates, such as DOM-bound Fe.^53,54^ The consumption of H_2_O_2_ would increase δ^18^O_H_2_O_2__ through the hypothesized normal KIE. Experiments with commercial H_2_O_2_ in the presence of DOM isolates corroborated a H_2_O_2_ consumption that elevated δ^18^O_H_2_O_2__ (Figure 3a), proportionally to the amount consumed (see details in Figure S5d). This explains the SRHA sample’s lowest H_2_O_2_ yield and highest δ^18^O_H_2_O_2__ among the isolates (Figure S9a,b, respectively). However, in Δ^18^O_H_2_O_2__, representing the relative difference in δ^18^O_H_2_O_2__ values between two pH levels, the impact of isotope fraction from side reactions cancels out, enabling quantification of the phenolic and olefinic fractions. If compounds exhibiting pH-dependent H_2_O_2_ consumption are present (e.g., transition metals in natural waters), they may bias Δ^18^O_H_2_O_2__. Therefore, H_2_O_2_ consumption and the corresponding changes in δ^18^O_H_2_O_2__ for the target water matrix at both pH levels should be evaluated using commercial H_2_O_2_ prior to Δ^18^O_H_2_O_2__ analysis.

## 4 Implications

A novel approach has been developed for determining the proportions of olefinic and phenolic DOM moieties based on isotopic analysis of H_2_O_2_ formed during ozonation. A contrasting pH-dependent δ^18^O_H_2_O_2__ evolution was observed for olefins and phenols for a range of model compounds. An empirical Δ^18^O_H_2_O_2__-*f*_P_ correlation equation, derived from mixture matrices, was applied to DOM isolates, providing the first estimate of olefinic content in DOM. For the broader applicability and higher precision of this method, further refinement will be needed through inclusion of a wider range of model compound types and validation across diverse synthetic matrices and DOM samples.

Unlike conventional DOM characterization techniques, which typically focus on direct analysis of the matrix itself, the unique feature of this Δ^18^O_H_2_O_2__ tracer method lies in its analytical basis of ozone byproducts. This approach offers novel insights as it directly targets functional groups that are relevant for oxidation processes. Thus, it can complement other DOM characterization methods, such as various phenol determination methods, providing information on the content of olefinic moieties. This approach can be applied to conditions similar to practical ozonation processes and does not require isotopically labeled reagents. The compositional understanding of DOM provided by this study is expected to enable better interpretation of DBP formation behavior, including product distribution, potential toxicity, and sensitivity to treatment conditions. For example, olefinic moieties yield low-molecular-weight carbonyl compounds, whereas phenolic moieties form a broader range of products, including quinones and catechols,^7,9^ which are potentially more toxic DBPs and may also be less biodegradable during biological postfiltration following ozonation. Thus, estimating the olefinic/phenolic fractions in DOM can help predict DBP formation potential and ultimately allows to develop DBP mitigation strategies for oxidation processes. Furthermore, the developed isotopic approach has a potential for broader applications in other H_2_O_2_-generating reactions, such as enzymatic H_2_O_2_ formation via oxidases^55,56^ or photo/electrochemical H_2_O_2_ production,^57,58^ to clarify the reaction mechanisms and the oxygen sources of H_2_O_2_ (H_2_O, O_2_, or metabolic intermediates).

## Supplementary Material



## Notes

Chemicals, DOM isolates, ozone stock solution, δ^18^O_H_2_O_2__ determination, HPLC measurements, determination of δ^18^O of O_3_, compilation of the olefinic and phenolic compounds, determination of the method detection limit for δ^18^O_H_2_O_2__, total peroxide yields for olefins, variations in δ^18^O_H_2_O_2__ of commercial H_2_O_2_ upon different reactions, ozonation of cinnamic acid-phenol mixtures, ozonation of DOM isolates at different ozone doses (PDF)
