# Supplementary material for: Determining Olefinic and Phenolic Fractions in Dissolved Organic Matter by Ozonation with Stable Oxygen Isotope Analysis of H2O2
Source: Environ Sci Technol. 2026 Apr 21;60(17):13181–91. doi: 10.1021/acs.est.6c00499 (PMC13151040; doi:10.1021/acs.est.6c00499)
Supplement: Supplementary file 1 [file es6c00499_si_001.pdf]

Supplementary Information

Determining Olefinic and Phenolic Fractions in Dissolved Organic Matter by Ozonation with Stable Oxygen Isotope Analysis of H_2O_2

Seok Kim[1], Maria Lia Halder[1], Thomas B. Hofstetter[1,2]*, and Urs von Gunten[1,2,3]*

[1] Swiss Federal Institute of Aquatic Science and Technology (Eawag), 8600 Dübendorf, Switzerland

[2] Institute of Biogeochemistry and Pollutant Dynamics (IBP), ETH Zürich, 8092 Zürich, Switzerland

[3] School of Architecture, Civil and Environmental Engineering (ENAC), Ecole Polytechnique Fédérale Lausanne (EPFL), 1015 Lausanne, Switzerland

Submitted to

Environmental Science & Technology

***Corresponding Authors:**
urs.vongunten@eawag.ch (U. von Gunten), thomas.hofstetter@eawag.ch (T. B. Hofstetter)

This Supporting Information contains 19 pages, including 4 supplementary texts (Texts S1–S4), 2 tables (Tables S1–S2), and 9 figures (Figures S1–S9).

Text S1. Chemicals, DOM isolates, and ozone stock solution

The following chemicals were used for model compound solutions: 3-buten-2-ol (97%, Sigma-Aldrich), *trans*-cinnamic acid (≥ 99%, Sigma-Aldrich), acrylic acid (99%, Sigma-Aldrich), methacrylic acid (99%, Sigma-Aldrich), sorbic acid (≥ 99%, Fluka), and *trans*-dichloroethylene (98%, Sigma-Aldrich), phenol (99.5%, Thermo Scientific), 4-nitrophenol (99.5%, Fluka), 4-chlorophenol (99.5%, Fluka), *p*-cresol (≥ 98.0%, Fluka), 4-methoxyphenol (99%, Sigma-Aldrich), hydroquinone (≥ 99%, Sigma-Aldrich), catechol (≥ 99.0%, Merck), dimethyl sulfoxide (≥ 99.9%, Sigma-Aldrich), sodium phosphate dibasic dihydrate (≥ 98%, Sigma-Aldrich), sodium phosphate monobasic monohydrate (99%, Sigma-Aldrich), sodium hydroxide (≥ 98%, Sigma-Aldrich), and phosphoric acid (≥ 85% w/w, Sigma-Aldrich). The following chemicals were used as analytical reagents: potassium iodide (≥ 99%, Merck), ammonium molybdate tetrahydrate (≥ 99.0%, Sigma-Aldrich), potassium hydrogen phthalate (≥ 99.5%, Merck), hydrogen peroxide solution (30% w/w, Sigma-Aldrich), sodium hypochlorite solution (10-15%, Sigma-Aldrich), ascorbic acid (99.0 - 100.5%, Merck), formaldehyde solution (37% w/w, Sigma-Aldrich), formic acid (≥ 98%, Sigma-Aldrich), and benzaldehyde (≥ 99.5%, Sigma-Aldrich). All aqueous solutions were prepared with ultra-purified water with a resistivity of > 18.2 MΩ·cm (Arium® pro Ultrapure Water Systems, Sartorius AG).

Three natural organic matter (NOM) isolates were obtained from the International Humic Substances Society (IHSS): Suwannee River II Standard Humic Acid (SRHA, 2S101H), Suwannee River II Standard Fulvic Acid (SRFA, 2S101F), and Upper Mississippi River Aquatic Natural Organic Matter (UMRNOM, 1R110N). Upon dissolution of NOM in water, it becomes dissolved organic matter (DOM), which is used in the manuscript.

Ozone stock solutions (1.6-1.9 mM) were prepared by generating ozone-containing oxygen gas by an ozone generator (BMT 803 BT, BMT Messtechnik) from pure oxygen (Carbagas, 99.995%), which was purged into ice-cooled ultra-purified water. The ozone concentration was determined spectrophotometrically at 260 nm with a molar absorption coefficient of 3200 $M^{-1}cm^{-1}$ (Carry 100, Varian).[1]

Text S2. $\delta^{18}O_{H_2O_2}$ determination

The $\delta^{18}O$ of H_2O_2 ($\delta^{18}O_{H_2O_2}$) was determined based on the method established in a previous study, with modifications to enhance precision in complex matrices.[2] Six procedural steps were carried out as follows:

1) H_2O_2 formation by ozonation: Ozonation was carried by spiking an aliquot of an ozone stock solution to a solution containing the target compounds in a 100 mL serum bottle under stirring (crimp top, Sigma-Aldrich) to achieve a specific ozone dose in a total volume of 100 mL. Two pH conditions (pH 3 and 7, 10 mM phosphate buffer) were used with 33 mM DMSO as a hydroxyl radical (·OH) scavenger. The ozone doses and model compound concentrations are provided in Table S1. For pH 7 samples, acidification was performed within 10 seconds after ozonation to stabilize H_2O_2. A blank sample was prepared for each sample solution by adding an equivalent volume of pure water instead of the ozone stock solution to the same matrix. 10 mL aliquots were collected from each sample for H_2O_2 quantification and compound consumption analysis.
2) Residual O_2 removal: 90 mL of the samples in the serum bottle, sealed with a rubber stopper and aluminum crimp cap, were purged with N_2 gas (99.999%) through a needle for 15 min to remove residual dissolved oxygen.
3) Sample transfer: three times 19.7 mL of each H_2O_2-containing sample was transferred to three 20 mL headspace crimp vials inside an anaerobic glovebox under N_2 atmosphere (O_2 < 0.1 ppm, UNIlab 2000, M. Braun) and sealed with crimp caps to maintain an oxygen-free environment.
4) Transformation of H_2O_2 to O_2: 460 µL of 1.49 M NaOCl and 40–120 µL of 2M NaOH were simultaneously injected into the cramped vial through a gas-tight Hamilton syringe. The volume of the NaOH solution was adjusted based on pH monitoring to achieve pH 7 after the injection (using the residual sample solution after the sample transfer step). After gently shaking the vial, 460 µL of a 0.75 M ascorbic acid solution (pH 0.8, adjusted by phosphoric acid) was injected to quench residual chlorine and lower the final pH to ~3. The NaOH, NaOCl, and ascorbic acid solutions were prepared in 10 mL headspace crimp vials, sealed, and purged with N_2. The O_2 conversion rate was evaluated by measuring the dissolved oxygen using a needle-type oxygen microsensor (NTH-PSt7, PreSens Precision Sensing GmbH) and comparing it to the blank sample.
5) Transfer of O_2 to the headspace: A headspace was created in the vial by replacing 3 mL of the sample solution with N_2 using a gas-tight syringe under an overpressure of 1.2 bar while holding

the vials upside down. The vials were placed on an orbital shaker for 30 min at 200 rpm to facilitate the transfer of O_2 to the gas phase.

6) δ^{18}O measurement: δ^{18}O of O_2 in the sample vials was analyzed by gas chromatography-isotope ratio mass spectrometry (GC/IRMS), consisting of a GC coupled via a Conflo IV interface to a Delta V Plus isotope ratio mass spectrometer. A 1500 μL headspace sample was single-injected into the split injector with He as carrier gas (99.999%) and a split flow of 40 mL/min. The detailed GC configuration and measurement conditions are described in previous studies.[2, 3] δ^{18}O values were determined from ratios of peak areas of masses 32 and 34, referenced against O_2 gas pulses introduced at the beginning of each chromatogram (99.995%, 2.98 ± 0.15 V peak height, σ δ18O = 0.07 ‰, n =221). The δ^{18}O value of the reference gas was calibrated against O_2 peaks from on-column injections of ambient air (70 μL), assuming a constant δ^{18}O of 23.88 ‰.[4]

Text S3. HPLC measurements

The concentrations of phenol and benzaldehyde in the samples before and after ozonation were measured by high-performance liquid chromatography coupled to a diode array detector (HPLC-DAD, Ultimate 3000, Thermo Scientific) after 10-fold dilution. The separation was performed on a Cosmosil 5C18-MS-II (3.0x100 mm) HPLC column with an eluent of 40% methanol/60% 10 mM H_3PO_4 at a flow rate of 0.8 mL/min. Detection wavelengths were set at 273 nm for phenol and 250 nm for benzaldehyde. The method was linear over the measuring range 2.5–30 μM ($R^2 > 0.999$) for both compounds. The concentrations of the analytes were significantly higher than the limit of quantitation with signal to noise ratios > 10.

Text S4. Determination of $\delta^{18}O$ of O_3

$\delta^{18}O$ of O_3 in an O_3 stock solution was determined by excluding the contribution from dissolved O_2. Since the O_3 stock solution contains both O_2 and O_3, isolating $\delta^{18}O$ of O_3 alone is challenging.[2] To address this, 5 mL of the stock solution was sampled into two reagent types to discriminate the O_2 effect: (i) 15 mL of 0.4 mM cinnamic acid with 10 mM phosphoric acid (pH 3) and (ii) 15 mL of 10 mM NaOH (pH 12). The reagent (i) reacts only with O_3, leaving dissolved O_2 unaffected. An acidic condition prevents potential O_2 generation from H_2O_2 decomposition. In contrast, reagent (ii) promotes the reaction between O_3 and OH^-, transforming all O_3 to O_2 (an 80% conversion ratio was confirmed by measuring dissolved oxygen). Both reagents were prepared in sealed 20 mL headspace crimp vials, which were purged with N_2 before the reaction. A gas-tight syringe was used to inject the O_3 stock solution into each vial. The resulting two samples were analyzed using GC/IRMS, with the results shown in Figure S2. The O_2 sample from reagent (i) showed 7.7‰ and a peak area of 30.3 V·s for $\delta^{18}O_{O_2}$ and a mass 32 (A_{O_2}), respectively. The $O_2 + O_3$ sample from reagent (ii) showed 20‰ and a peak area of 79.1 V·s for $\delta^{18}O_{O_2+O_3}$ and a mass 32 ($A_{O_2+O_3}$), respectively. Using the following correction equation to exclude the O_2 effect from the O_2+O_3 sample,[3, 5] $\delta^{18}O$ of O_3 was determined to be 28‰:

$$\delta^{18}O_{O_3} = \frac{\delta^{18}O_{O_2+O_3} \cdot A_{O_2+O_3} - \delta^{18}O_{O_2} \cdot A_{O_2}}{A_{O_2+O_3} - A_{O_2}}$$

Table S1. Compilation of the selected olefinic and phenolic compounds. pK_a values, second-order rate constants for their reactions with O_3 and ·OH, and ozonation conditions for model compounds.

	**Olefinic compounds**						**Phenolic compounds**						
Name	3-Buten-2-ol	Cinnamic acid	Acrylic acid	Methacrylic acid	Sorbic acid	*trans*-dichloro-ethene	Phenol	4-Nitro	4-Chlro	4-Methyl	4-Methoxy	4-hydroxy (Hydroquinone)	2-hydroxy (Catechol)
pK_a	-	4.44[a]	4.25[a]	4.70[a]	4.75[a]	-	9.98[a]	7.15[a]	9.38[a]	10.30[a]	10.21[a]	9.25[a]	9.96[a]
k_{O_3}, protonated (M^{-1} s^{-1})	7.9×10^{4}[a]	5.0×10^{4}[a]	2.8×10^{4}[a]	1.5×10^{5}[a]	3.2×10^{5}[a]	6.5×10^{3}[a]	1.3×10^{3}[a]	<50[a]	6.00×10^{2}[a]	3.0×10^{4}[b]	4.7×10^{5}[b]	N/A[c]	N/A[c]
k_{O_3}, deprotonated (M^{-1} s^{-1})		3.8×10^{5}[a]	1.6×10^{5}[a]	3.7×10^{6}[a]	9.6×10^{5}[a]		1.4×10^{9}[a]	1.7×10^{7}[a]	6.0×10^{8}[a]	3.2×10^{9}[b]	1.1×10^{10}[b]	N/A[c]	N/A[c]
$k_{\cdot OH}$ (M^{-1} s^{-1})	3.57×10^{10}[d]	8.1×10^{9}[e]	8.4×10^{9}[e]	1.1×10^{10}[f]	8.1×10^{9}[e]	6.2×10^{9}[e]	1.4×10^{10}[e]	3.8×10^{9}[e]	7.6×10^{9}[e]	1.2×10^{10}[e]	2.6×10^{10}[e]	5.2×10^{9}[e]	1.1×10^{10}[e]
**Ozonation conditions**													
[Compound] (μM)	300	200	600	600	300	1200	800	1200	1000	800	450	800	800
[O_3] (μM)	80	40-90	120	120	80	300	260	390	260	260	220	260	260
[Comp.]/[O_3]	3.8	2.2-5	5.0	5.0	3.8	4	3.1	3.1	3.8	3.1	2.0	3.1	3.1

[a]von Sonntag and von Gunten.[1] [b]Tentscher et al.[6] [c]$k_{O_3,app}$ values were measured at pH 3 and 7 for those compounds.[1] [d]Du et al.[7] [e]Buxton et al.[8] [f]Schöne et al.[9]

Table S2. Summary of H_2O_2 formation data. Number of replicates, H_2O_2 yields, organic peroxide yields, relative O_2 conversion efficiency, $\delta^{18}O_{H_2O_2}$, and $\Delta^{18}O_{H_2O_2}$ for model compounds.

	**Olefinic compounds**						**Phenolic compounds**						
Name	3-Buten-2-ol	Cinnamic acid	Acrylic acid	Methacrylic acid	Sorbic acid	*trans*-dichloro-ethene	Phenol	4-Nitro	4-Chlro	4-Methyl	4-Methoxy	4-hydroxy (Hydroquinone)	2-hydroxy (Catechol)
Experimental replicate	1	3	4	3	3	2	4	2	3	3	3	2	2
**H_2O_2 yields by the $^1O_2$ method**													
H_2O_2 yield at pH 3 (%)	92.6 ± 4.8	91.0 ± 6.2	42.8 ± 2.6	37.1 ± 2.1	96.3 ± 5.7	8.3 ± 0.2	29.8 ± 4.8	3.7 ± 0.2	9.8 ± 0.7	20.9 ± 1.0	10.9 ± 0.4	11.3 ± 0.3	8.8 ± 0.4
H_2O_2 yield at pH 7 (%)	90.7 ± 1.8	86.1 ± 2.7	41.5 ± 1.7	42.4 ± 2.4	91.9 ± 3.1	7.0 ± 0.4	14.0 ± 1.5	1.0 ± 0.0	6.9 ± 0.3	6.0 ± 0.3	8.3 ± 0.3	10.5 ± 0.3	6.1 ± 0.3
**H_2O_2/organic peroxide yields by the Allen's reagent method**													
H_2O_2 yield at pH 3 (%)	40.7 ± 0.8	96.7 ± 2.8	22.8 ± 2.7	31.9 ± 1.8	85.0 ± 0.6	10.4 ± 0.2	N/A						
Organic peroxide yield at pH 3 (%)	57.9 ± 1.0	4.2 ± 0.6	57.5 ± 2.8	48.9 ± 2.8	13.5 ± 1.3	0.3 ± 0.1							
H_2O_2 yield at pH 7 (%)	52.0 ± 0.2	95.7 ± 0.7	22.7 ± 2.2	44.2 ± 2.4	94.2 ± 2.2	9.7 ± 0.4							
Organic peroxide yield at pH 7 (%)	46.6 ± 0.3	4.1 ± 0.7	56.0 ± 1.8	41.5 ± 1.3	2.7 ± 0.3	0.2 ± 0.0							
**H_2O_2-to-O_2 conversion**													
O_2 conversion at pH 3 (%)	100 ± 1	88 ± 12	86 ± 4	103 ± 4	87 ± 5	87 ± 3	102 ± 2	90 ± 5	85 ± 2	86 ± 6	111 ± 8	88 ± 3	101 ± 3
O_2 conversion at pH 7 (%)	104 ± 5	99 ± 2	100 ± 3	99 ± 2	92 ± 9	101 ± 9	88 ± 0.7	76 ± 8	97 ± 4	100 ± 12	97 ± 13	89 ± 2	96 ± 1
**Isotope signatures**													
Measurement replicate	3	9	12	9	9	5	12	5	8	9	8	6	6
$\delta^{18}O_{H_2O_2}$ at pH 3 (‰)	58.5 ± 1.3	60.5 ± 0.5	49.4 ± 1.0	55.6 ± 2.0	55.1 ± 1.2	93.7 ± 1.3	58.4 ± 1.5	57.5 ± 4.4	52.5 ± 1.4	62.4 ± 0.8	47.4 ± 2.6	44.5 ± 1.0	44.3 ± 0.9
$\delta^{18}O_{H_2O_2}$ at pH 7 (‰)	58.9 ± 0.5	61.1 ± 0.3	51.4 ± 1.5	58.1 ± 1.8	59.6 ± 1.3	103 ± 1.0	51.0 ± 1.1	53.5 ± 5.0	48.9 ± 1.3	48.2 ± 1.2	45.7 ± 1.1	40.5 ± 0.2	38.3 ± 1.5
$\Delta^{18}O_{H_2O_2}$ (‰)	0.4 ± 1.4	0.6 ± 0.6	2.0 ± 1.8	2.4 ± 2.7	4.4 ± 1.8	9.0 ± 1.4	-7.4 ± 1.9	-4.0 ± 6.7	-3.6 ± 1.9	-14.2 ± 1.4	-1.6 ± 2.8	-4.0 ± 1.0	-6.0 ± 1.8

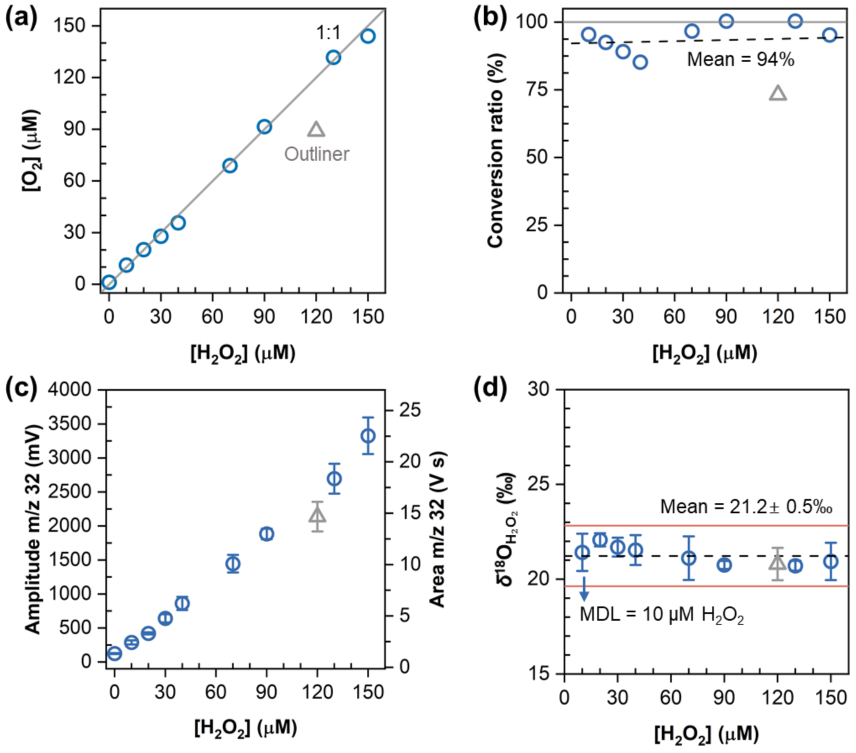

Figure S1. Determination of the method detection limit (MDL) for $\delta^{18}O_{H_2O_2}$ for the commercial H_2O_2 samples. H_2O_2-to-O_2 conversion by chlorination: (a) formed O_2 as a function of H_2O_2 (O_2 quantified by the oxygen sensor), (b) conversion efficiency of H_2O_2 to O_2. GC-IRMS measurement of transformed O_2 as a function of the H_2O_2 concentration: (c) amplitude and area of m/z 32 peaks and (d) $\delta^{18}O_{H_2O_2}$. The black dashed lines represent the mean values, the gray solid line represents a 100% conversion ratio, and the red solid lines represent the precision error of GC-IRMS for ^{18}O detection.[10] The data point at 120 µM H_2O_2 (grey triangle) is considered an outlier, assumably ascribed to O_2 leakage while using the oxygen sensor. The MDL is determined according to the moving mean procedure.[11] The mean $\delta^{18}O_{H_2O_2}$ value aligns with a previous study (21.9 ± 0.7‰) while showing a higher conversion ratio and lower MDL than the previously reported 90% conversion and 12 µM MDL (panel (d)).[2] Conditions: [phosphate buffer] = 10 mM (pH 3), [DMSO] = 5 mM, [H_2O_2] = 0, 10, 20, 30, 40, 70, 90, 120, 130, and 150 µM.

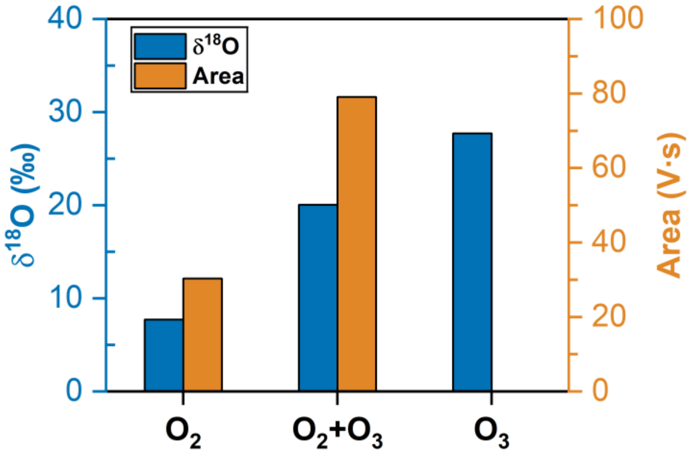

Figure S2. Determination of δ^{18}O of O_3. δ^{18}O of O_3 in the O_3 stock solution was analyzed by compensation of the O_2 effect (O_2) from the stock solution containing O_2 and O_3 (O_2+O_3). See Text S4 for details.

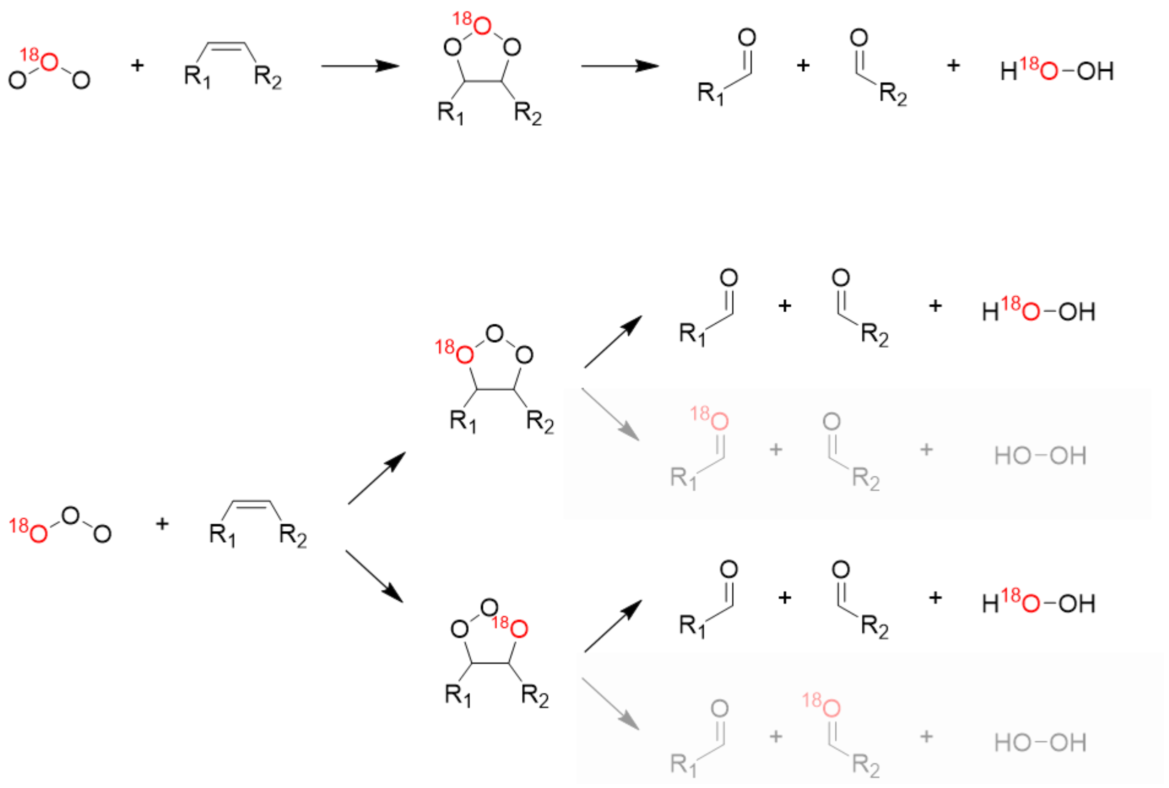

Figure S3. Fractionation of H_2O_2 generation via the Criegee reaction with O_3 isopologues containing an ^{18}O. While ^{16}O-^{18}O-^{16}O follows one pathway that produces heavy H_2O_2 (^{18}O-containing), ^{18}O-^{16}O-^{16}O has other pathways generating light H_2O_2 (without ^{18}O). However, the preferential bond cleavage of ^{16}O-^{16}O in the Criegee ozonide drives this reaction also to heavy H_2O_2 formation. This leads to ^{18}O enrichment in H_2O_2 during the Criegee reaction. The black O atom represents ^{16}O.[2]

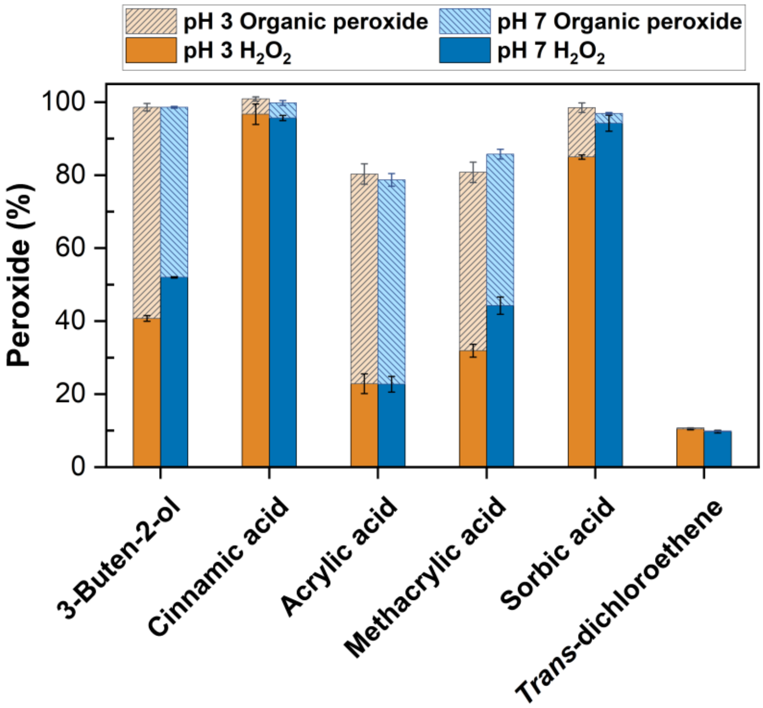

Figure S4. Total peroxide (H_2O_2 and organic peroxides) yields for the ozone reaction with olefinic model compounds, determined by the Allen's reagent method.

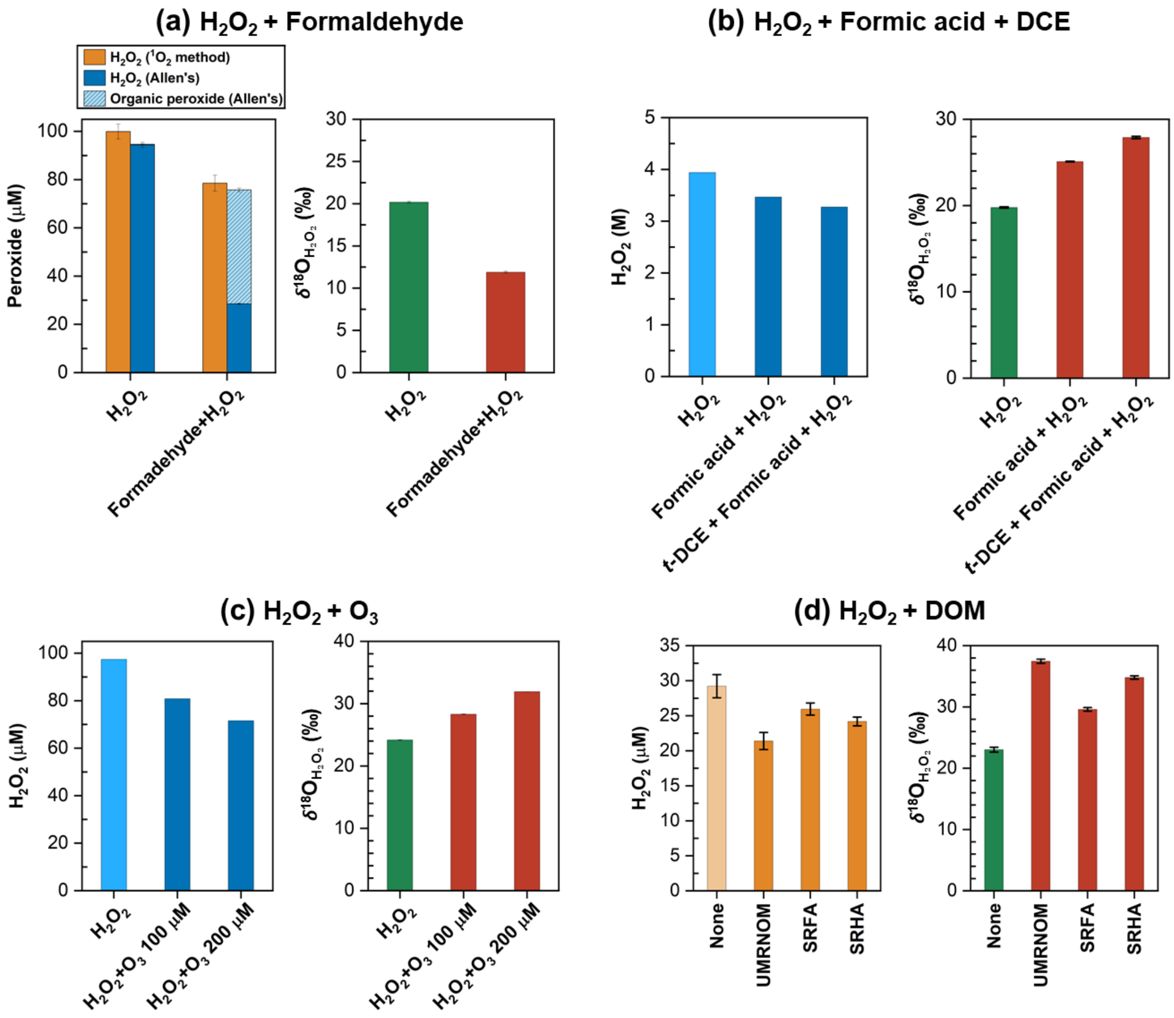

Figure S5. Variations in $\delta^{18}O_{H_2O_2}$ of commercial H_2O_2 upon reaction with different compounds. (a) Reaction with formaldehyde, which forms an equilibrium with an organic peroxide (hydroxymethyl-hydroperoxide), resulting in decreased $\delta^{18}O_{H_2O_2}$ due to preferential bond formation with ^{18}O in the organic peroxide. Conditions: $[H_2O_2]$ = 100 μM, [formaldehyde] = 15 mM, [phosphate buffer] = 10 mM, pH 7, reaction time = 40 min, T = room temperature. For the H_2O_2-to-O_2 conversion, a relatively lower chlorine dose (0.4 mM) was used to capture the $\delta^{18}O$ of H_2O_2, but minimizing the O_2 conversion from the organic peroxide. (b) Reaction with formic acid produces performic acid, which undergoes self-decomposition and reacts with *trans*-dichloroethene (*t*-DCE), reducing the H_2O_2 concentration.[12, 13] This leads to higher $\delta^{18}O_{H_2O_2}$ due to preferential ^{16}O-^{16}O bond cleavage in the hydroperoxide group of performic acid. Conditions: $[H_2O_2]$ = 4 M, [formic acid] = 4 M, [sulfuric acid] = 1 M, reaction time = 6 h, T = 40°C. (c) Reaction of H_2O_2 with O_3 at varying doses, where the preferential ^{16}O reaction with O_3 in H_2O_2 leads to an increased $\delta^{18}O_{H_2O_2}$. Conditions: $[H_2O_2]$ = 100 μM, [phosphate buffer] = 10 mM, pH 7, [DMSO] = 1 mM, reaction time = 10 min, T = room temperature. (d) Reaction in DOM isolate-containing solutions, resulting in increased $\delta^{18}O_{H_2O_2}$, also

due to preferential ^{16}O reactions in H_2O_2. Conditions: $[H_2O_2]$ = 30 μM, [DOC] = 45 mg C L^{-1}, [phosphate buffer] = 10 mM, pH 3, [DMSO] = 33 mM, reaction time = 3 h, T = room temperature.

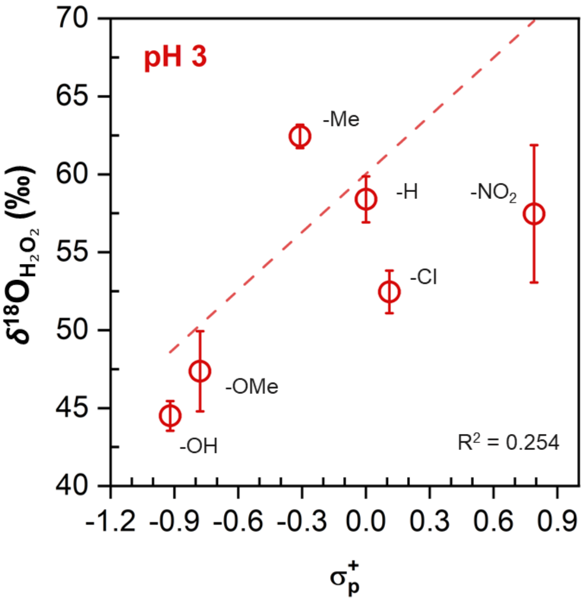

Figure S6. Correlation between $\delta^{18}O_{H_2O_2}$ from ozonation of para-substituted phenols at pH 3 and their Hammett constants. The $\delta^{18}O_{H_2O_2}$ values are taken from phenols at pH 7 in Figure 1b.

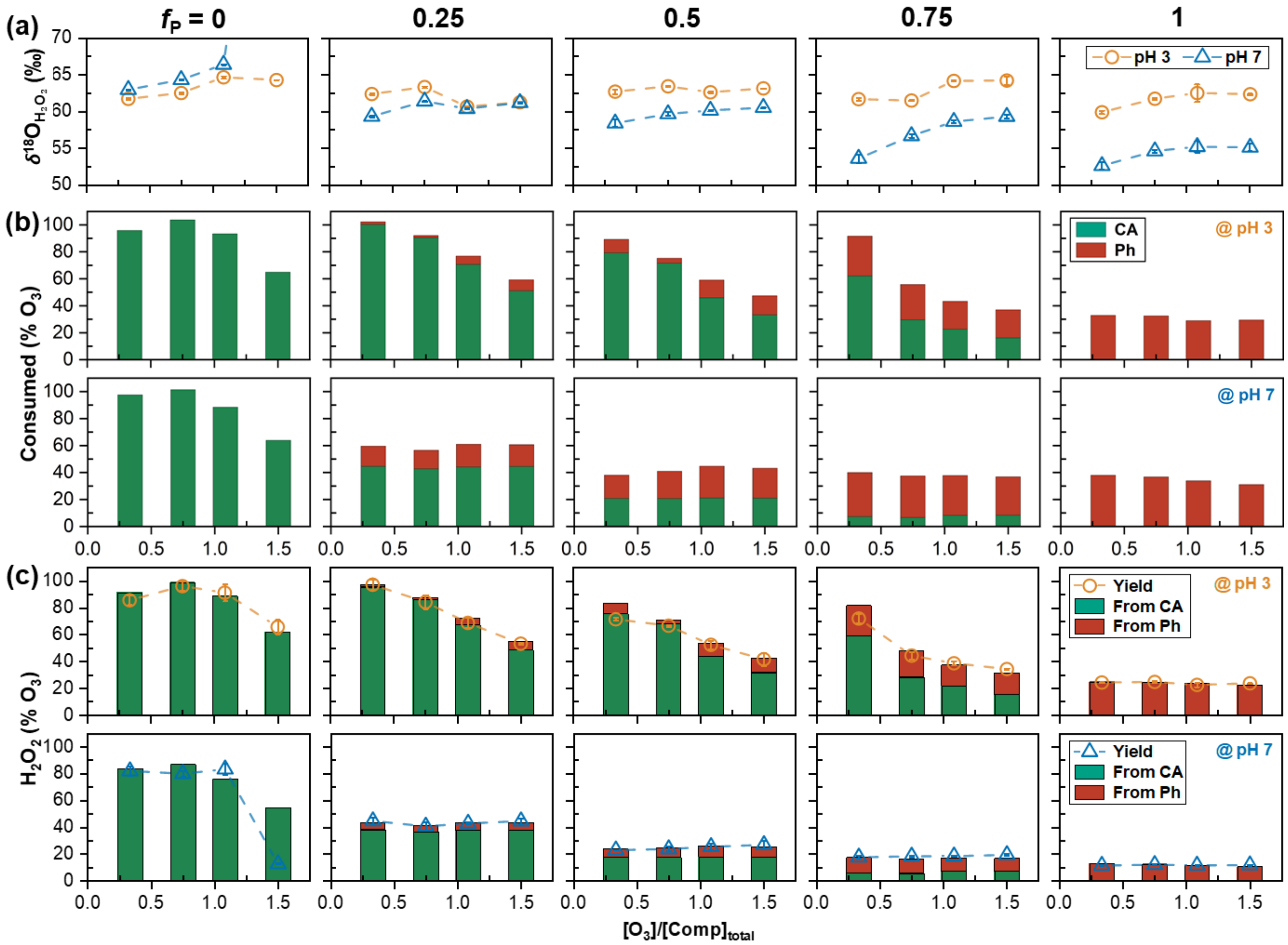

Figure S7. Ozonation of cinnamic acid (CA)-phenol (Ph) mixtures with varying molar phenolic fraction (f_P) as a function of the specific molar O_3 dose. (a) $\delta^{18}O_{H_2O_2}$, (b) relative consumed compound (% of O_3 dose), and (c) H_2O_2 yields (% of O_3 dose). For H_2O_2 yields, the dashed lines indicate H_2O_2 quantified by the $^1O_2$ method and the bars present estimated H_2O_2 contributions from each compound, which were calculated by $[CA]_{consumed} \times 0.95$ (pH 3) or 0.86 (pH 7) + $[Ph]_{consumed} \times$ 0.77 (pH 3) or 0.34 (pH 7). For the H_2O_2 yields of CA and Ph, the average yields from $f_p = 0$ and f_P = 1 are used, respectively, to calculate the relative contributions. Conditions: $[compounds]_{total}$ = 300 μM, $[O_3]$/[compounds] = 0.3, 0.75, 1.1, 1.5 mol mol^{-1}, [phosphate buffer] = 10 mM (pH 3 or pH 7), [DMSO] = 33 mM.

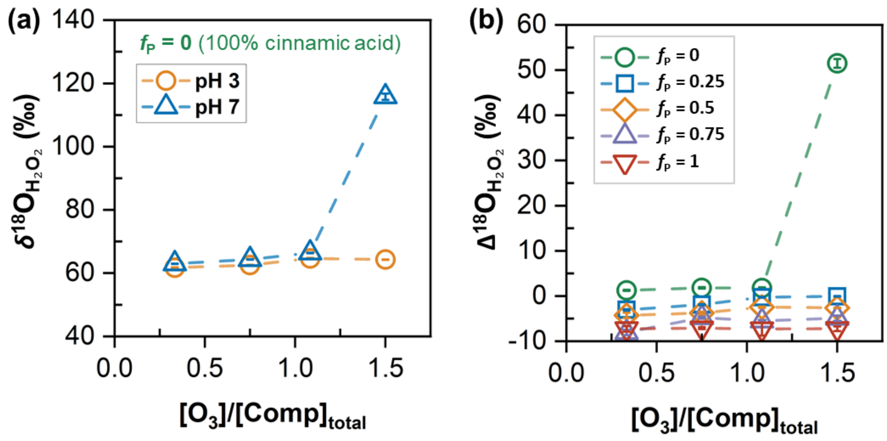

Figure S8. Ozonation of (a) cinnamic acid and (b) cinnamic acid-phenol mixtures as a function of the specific molar O_3 doses. An increase in $\delta^{18}O_{H_2O_2}$ at pH 7 and $\Delta^{18}O_{H_2O_2}$ with an excess molar O_3 dose ($[O_3]$/[cinnamic acid] = 1.5 mol mol^{-1}) was observed with $f_P = 0$ (100% cinnamic acid). Conditions: $[\text{Compounds}]_{total}$ = 300 µM, $[O_3]$/[compounds] = 0.3, 0.75, 1.1, 1.5 mol mol^{-1}, [phosphate buffer] = 10 mM (pH 3 or pH 7), [DMSO] = 33 mM.

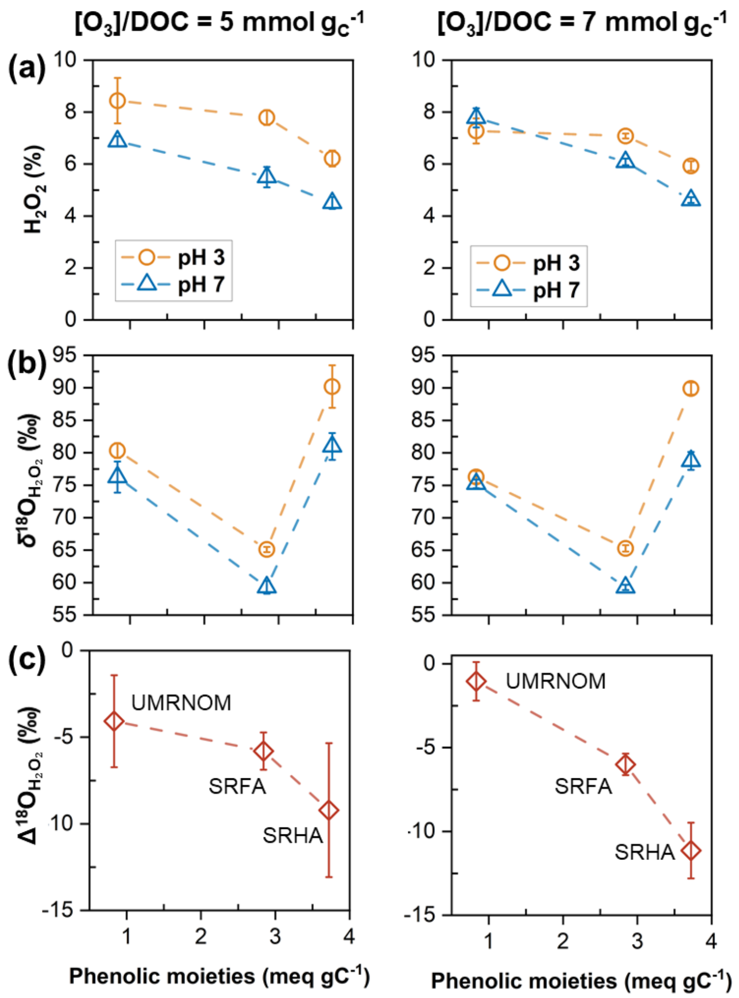

Figure S9. Ozonation of DOM isolates for two specific ozone doses at pH 3 and 7. (a) Molar H_2O_2 yields (% relative to consumed O_3), (b) $\delta^{18}O_{H_2O_2}$, and (c) $\Delta^{18}O_{H_2O_2}$ as a function of the phenolic content of the DOM, reported by IHSS.[14] Conditions: [DOC] = 45 mg L^{-1}, $[O_3]$/DOC = 5 and 7 mmol g_C^{-1}, [phosphate buffer] = 10 mM (pH 3 or pH 7), [DMSO] = 33 mM.

References

(1) von Sonntag, C.; von Gunten, U. *Chemistry of ozone in water and wastewater treatment-From Basic Principles to Applications*; IWA publishing, 2012.

(2) Houska, J.; Stocco, L.; Hofstetter, T. B.; von Gunten, U. Hydrogen peroxide formation during ozonation of olefins and phenol: mechanistic insights from oxygen isotope signatures. *Environmental Science & Technology* **2023**, *57* (47), 18950-18959.

(3) Bopp, C. E.; Bolotin, J.; Pati, S. G.; Hofstetter, T. B. Managing argon interference during measurements of 18O/16O ratios in O2 by continuous-flow isotope ratio mass spectrometry. *Analytical and Bioanalytical Chemistry* **2022**, *414* (20), 6177-6186.

(4) Barkan, E.; Luz, B. High-precision measurements of 17O/16O and 18O/16O of O2 and O2/Ar ratio in air. *Rapid communications in mass spectrometry* **2003**, *17* (24), 2809-2814.

(5) Pati, S. G.; Bolotin, J.; Brennwald, M. S.; Kohler, H. P. E.; Werner, R. A.; Hofstetter, T. B. Measurement of oxygen isotope ratios (18O/16O) of aqueous O2 in small samples by gas chromatography/isotope ratio mass spectrometry. *Rapid Communications in Mass Spectrometry* **2016**, *30* (6), 684-690.

(6) Tentscher, P. R.; Bourgin, M.; von Gunten, U. Ozonation of para-substituted phenolic compounds yields p-benzoquinones, other cyclic α, β-unsaturated ketones, and substituted catechols. *Environmental science & technology* **2018**, *52* (8), 4763-4773.

(7) Du, B.; Feng, C.; Zhang, W.; Mu, L. Theoretical study on the mechanism for the reaction of OH with CH2CHCH2CH2OH. *Chemical Physics* **2010**, *367* (1), 52-61.

(8) Buxton, G. V.; Greenstock, C. L.; Phillips Helman, W.; Ross, A. B. Critical review of rate constants for reacitons of hydrated electrons. *J. Phys. Chem. Ref. Data;(United States)* **1988**, *17* (2).

(9) Schöne, L.; Schindelka, J.; Szeremeta, E.; Schaefer, T.; Hoffmann, D.; Rudzinski, K. J.; Szmigielski, R.; Herrmann, H. Atmospheric aqueous phase radical chemistry of the isoprene oxidation products methacrolein, methyl vinyl ketone, methacrylic acid and acrylic acid–kinetics and product studies. *Physical Chemistry Chemical Physics* **2014**, *16* (13), 6257-6272.

(10) Elsner, M.; Jochmann, M. A.; Hofstetter, T. B.; Hunkeler, D.; Bernstein, A.; Schmidt, T. C.; Schimmelmann, A. Current challenges in compound-specific stable isotope analysis of environmental organic contaminants. *Analytical and bioanalytical chemistry* **2012**, *403*, 2471-2491.

(11) Jochmann, M. A.; Blessing, M.; Haderlein, S. B.; Schmidt, T. C. A new approach to determine method detection limits for compound-specific isotope analysis of volatile organic compounds. *Rapid Communications in Mass Spectrometry: An International Journal Devoted to the Rapid Dissemination of Up-to-the-Minute Research in Mass Spectrometry* **2006**, *20* (24), 3639-3648.

(12) Cogliano, T.; Russo, V.; Turco, R.; Santacesaria, E.; Di Serio, M.; Salmi, T.; Tesser, R. Revealing the role of stabilizers in H2O2 for the peroxyformic acid synthesis and decomposition kinetics. *Chemical Engineering Science* **2022**, *251*, 117488.

(13) Nabintu Kajoka, C.; Gasperi, J.; Brosillon, S.; Caupos, E.; Mebold, E.; Oliveira, M.; Rocher, V.; Chebbo, G.; Le Roux, J. Reactivity of Performic Acid with Organic and Inorganic Compounds: From Oxidation Kinetics to Reaction Pathways. *ACS ES&T Water* **2023**, *3* (9), 3121-3131.

(14) IHSS. *International Humic Substances Society*. https://humic-substances.org/ (accessed 2024-12-03).
